# Ferrous Selenide Stabilized Black Phosphorus Heterojunction Sonosensitizer for MR Imaging-Guided Sonodynamic Therapy of Bladder Cancer

**DOI:** 10.34133/bmr.0014

**Published:** 2024-03-27

**Authors:** Sicheng Wu, Guanlin Li, Wenrui Ouyang, Yuan Tian, Shujue Li, Wenqi Wu, Hongxing Liu

**Affiliations:** ^1^ Guangdong Provincial Key Laboratory of Urology, Guangdong Engineering Research Center of Urinary Minimally invasive surgery Robot and Intelligent Equipment, Guangzhou Institute of Urology, Department of Urology, The First Affiliated Hospital of Guangzhou Medical University, Guangzhou Medical University, Guangzhou 510120, China.; ^2^ The Second Affiliated Hospital of Guangzhou Medical University, Guangzhou Medical University, Guangzhou, China.

## Abstract

It is urgent to develop an alternative dynamic therapy-based method to overcome the limited efficacy of traditional therapy methods for bladder cancer and the damage caused to patients. Sonodynamic therapy (SDT) has the advantages of high tissue penetration, high spatiotemporal selectivity, and being non-invasive, representing an emerging method for eradicating deep solid tumors. However, the effectiveness of SDT is often hindered by the inefficient production of reactive oxygen species and the nondegradability of the sonosensitizer. To improve the anti-tumor effect of SDT on bladder cancer, herein, a BP-based heterojunction sonosensitizer (BFeSe_2_) was synthesized by anchoring FeSe_2_ onto BP via P–Se bonding to enhance the stability and the effect of SDT. As a result, BFeSe_2_ showed great cytotoxicity to bladder cancer cells under ultrasound (US) irradiation. BFeSe_2_ led to a notable inhibition effect on tumor growth in subcutaneous tumor models and orthotopic tumor models under US irradiation. In addition, BFeSe_2_ could also enhance T2-weighted magnetic resonance imaging (MRI) to achieve monitoring and guide treatment of bladder cancer. In general, BFeSe_2_ sonosensitizer integrates MRI functions for precise treatment, promising great clinical potential for the theranostics of bladder cancer.

## Introduction

Bladder cancer, due to its high incidence rate and recurrence rate, is one of the most serious malignancies of the urogenital system globally [1]. There are about 430,000 newly diagnosed cases and more than 165,000 deaths each year, which has become a serious public health problem [2]. According to the newest European Association of Urology guidelines, the treatment of bladder cancer mainly includes surgical treatment, chemotherapy drug infusion, targeted drug therapy, etc. [3]. Although there are various treatment methods for bladder cancer, their effect on treating bladder cancer is limited and they still cause immense pain to patients [4]. Consequently, it has important clinical value and scientific significance to develop an effective treatment strategy for bladder cancer while alleviating the sufferings of the patient.

Sonodynamic therapy (SDT) is an emerging therapeutic approach that can realize reactive oxygen species (ROS)-inducing apoptosis or necrosis by utilizing the ultrasound (US) to activate the sonosensitizers that accumulate in tumor cells [5–10]. Compared with traditional treatment methods, SDT is considered one of the most efficient non-invasive cancer treatment methods [11,12]. The US could penetrate tens of centimeters of soft tissue without the need for endoscopy or other bladder interventions. However, conventional sonosensitizers, such as organic dyes (porphyrins) or TiO_2_-derived nanomaterials, usually have poor stability or low sonosensitive effect [10,13–17]. Thus, sonosensitizers with good biocompatibility, effective sonosensitivity, high stability, and a clear mechanism to expand the application of SDT need to be developed urgently.

Black phosphorus (BP) as a 2-dimensional semiconductor, has gained increasing attention in biomedical fields due to its good biocompatibility and biodegradability, and its non-toxic metabolites (phosphates and phosphonates) [18]. Recent studies have found that BP can exhibit cytotoxicity through the production of ROS when stimulated by the US, demonstrating that it has excellent sonosensitivity [19–23]. However, the surface of BP contains lone pair electrons that are easily oxidized when in contact with air and water, which limits the application of SDT. Conventional methods for stabilizing BP, such as surface modification and doping [24], require complex operations, and it is difficult to achieve the dual goals of stabilizing BP and applying BP as a high-resolution and efficient probe in tumor theranostics since BP itself lacks magnetic resonance imaging (MRI) ability. Although cystoscopy can accurately diagnose nonmuscle invasive bladder cancer, it will cause some pain to patients. Therefore, it is necessary to further construct a BP nanoplatform with imaging capabilities, which not only enhances SDT but also presents good diagnostic capabilities [25–27].

Ferrous selenide (FeSe_2_) is a transition metal chalcogenide with good paramagnetism and biocompatibility, which can be used for MRI [6,28,29]. However, individual FeSe_2_ nanoparticles are also prone to oxidation in air. In previous studies, we have proved that FeSe_2_ can be covalently connected to BP via P–Se bonds to improve their stability and photothermal properties [30].

**Fig. 1.**
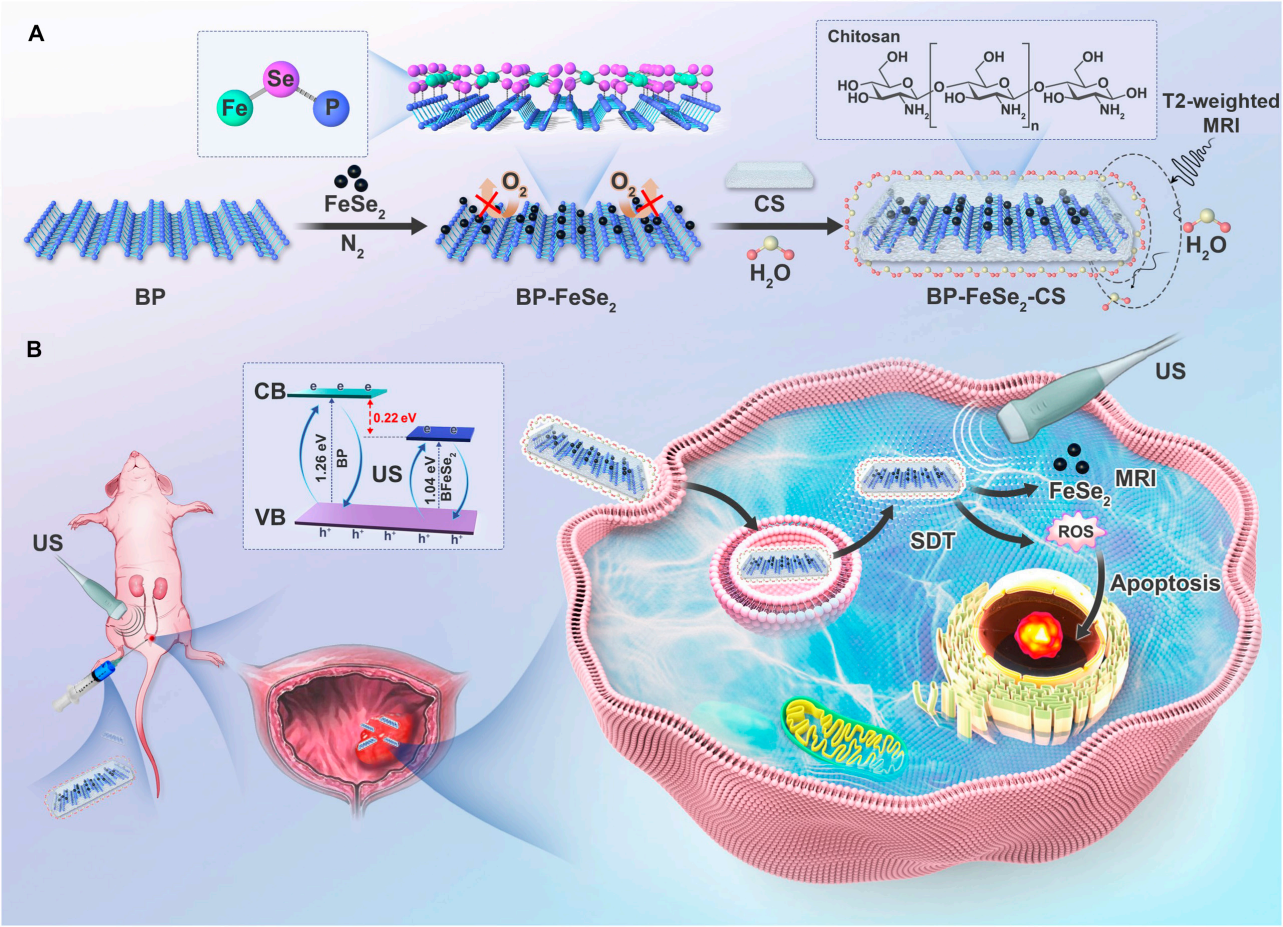
Schematic illustration of the BFeSe_2_ as an MRI-guided agent for SDT. (A) This schematic outlines the simple preparation of BP-FeSe_2_-CS (BFeSe_2_) and (B) reveals its mechanism of enhanced SDT for bladder cancer.

Herein, we hypothesized that the BP-FeSe_2_ heterostructures (BFeSe_2_) could improve the stability and sonosensitivity, to realize enhanced SDT for bladder cancer (Fig. 1A). Because FeSe_2_ has great paramagnetism, BFeSe_2_ is endowed with MRI function. Therefore, the advantage of BFeSe_2_ is that it could act as an efficient and biodegradable sonosensitizer and MRI reagent for precise treatment (Fig. 1B). We investigated and elucidated the mechanism of the photoelectron transfer process and the enhancement of sonodynamics efficiency. The band gap of BFeSe_2_ had been narrowed, making it easier to be excited by US. Then, the excited electrons on BFeSe_2_ were effectively captured and transferred through the FeSe_2_. Eventually, the electron–hole pair is effectively separated, thus enhancing ROS production. Therefore, SDT efficacy was enhanced through massive ROS generation, which induced bladder cancer cell apoptosis. Besides, the SDT effectively inhibited tumor proliferation in nude mice and caused no apparent toxicity. In addition, due to the superparamagnetism ability of FeSe_2_, BFeSe_2_ sonosensitizer was endowed with MRI function. In conclusion, BFeSe_2_ not only enhanced SDT efficacy for bladder cancer but also promised potential for MRI-guided precise treatment.

## Methods

### Materials and reagents

Anhydrous ethanol (C_2_H_6_O) was purchased from Guangzhou Chemical Reagent Factory. 2′,7′-Dichlorofluorescein diacetate (DCFH-DA), calcein acetyloxymethyl ester (calcein AM), propidium iodide (PI), and mitochondrial membrane potential detection kits were obtained from Beyotime Biotechnology (China). The ANNEXIN-APC/PI double staining kit and the Cell Counting Kit-8 (CCK-8) were purchased from Dongren Chemical Technology (Shanghai) Co., Ltd. The cell cycle kit was purchased from KaiJi Biotechnology Co., Ltd. The experimental water was Milli-Q secondary ultrapure water (18.2 MΩ·cm^−1^). The chemical reagents used in the experiment were analytical grade and had not been purified. MB49 cells were purchased from Beina Biotechnology Co., Ltd. MB49-Luc cell was purchased from Xiamen Immocell Biotechnology Co., Ltd. If not specified, all other chemicals are commercially available and used as received.

### Instrument

The high-speed centrifuge (Sorva II wx100+) and magnetic stirrer (KX-79-I) were used for the preparation of nanomaterials. Transmission electron microscopy (TEM) imaging was performed using JEM-2100 electron microscopy. X-ray photoelectron spectroscopy (XPS; Thermo Fisher Scientific K-Alpha) was used for chemical analysis. The Malvern Zeta Sizer Nano (Malvern Instruments) was applied to measure the Zeta potential. The U-4100 ultraviolet-visible spectrophotometer (Hitachi, Japan) was used to measure the absorption spectrum. A 1-MHz US instrument (NSE-UP-1M) was used to induce SDT. The absorbance of the CCK-8 method was measured in an enzyme-linked immunosorbent assay (RT6000, Rayto, USA). Fluorescence imaging experiments were performed using an inverted fluorescence microscope (Olympus Company). Cell apoptosis was measured using flow cytometry (BD FACSVERETM). The Living Animal Imaging System (IVIS Lumina III, US) was used for live imaging. The PathScope 4S tissue scanner was used for tissue slice scanning and analysis.

### Cells and animals

MB49 cells and MB49-Luc cells were cultured in a Dulbecco’s Modified Eagle Medium containing 10% fetal bovine serum at pH 7.4, 37 °C, and 5% CO_2_. Both cell experiments and animal experiments used logarithmic growth phase cells.

Balb/c nude mice (female, aged 6 to 8 weeks) were purchased from Guangdong Medical Experimental Animal Center. All animal experiments were carried out in compliance with the guidelines and regulations set forth by the Institutional Animal Care and Use Committee at Jennio Biotech Co., Ltd. Ethics number: #JENNIO-IACUC-2023-A031.

### Preparation of BP-CS

After grinding the blocky BP, 50 mg of BP powder was taken and dissolved in 200 ml of N-methylpyrrolidone (NMP). It was then peeled in an ice water bath with 960 W US for different times (8, 10, and 12 h) in an ultrasonic crusher. The stripped solution was centrifuged (5,000 rpm, 10 min) to separate the precipitate. Then, the BP nanosheets were obtained from the supernatant after centrifugation at 14,400 rpm for 20 min [31]. Chitosan (CS; 5 ml) was dispersed in 5 ml of BP aqueous solution with a concentration of 200 μg/ml, sonicated for 30 min, stirred for 4 h, centrifuged at 4,000 rpm at 4 °C for 30 min, and washed 2 times using the same method. The precipitate was dissolved in water to obtain BP-CS, which was then stored in the dark at 4 °C.

### Preparation of FeSe_2_-CS

In a 3-necked flask, 15 ml of oleylamine and 10 ml of 1-octadecene were combined and maintained at 120 °C under the N_2_ conditions. After 30 min, 1 mmol FeCl_2_·4H_2_O was added to the solution immediately and stirred for 30 min, and the temperature was kept constant. Under N_2_ conditions, 2 mmol selenium powder was added to 4 ml of oleylamine and heated until dissolved. Then, the dissolved substance was injected into the flask at a slow pace. Subsequently, the mixture was quickly heated to 150 °C and sustained for 30 min. Under N_2_ conditions, the reaction was cooled to approximately 25 °C. Then, an excess of cyclohexane was added to the solution and the reaction was centrifuged to obtain FeSe_2_. Finally, the FeSe_2_ was dissolved in anhydrous ethanol and stored under N_2_ conditions [28].

Excess polyacrylic acid (PAA) was dissolved in water, and 5 ml of PAA solution was slowly added to 1 ml of FeSe_2_ ethanol solution under US. After 30 min of US, the mixture was subjected to magnetic stirring for 6 h and centrifuged at 8,000 rpm for 15 min to remove excess ethanol and PAA, and then the precipitate was dissolved in water. CS was added to the FeSe_2_-PAA aqueous solution, which was sonicated for 30 min, stirred overnight at room temperature, centrifuged at 14,400 rpm for 15 min, dissolved in water to obtain FeSe_2_-CS, and stored under N_2_ conditions.

### Preparation of BP-FeSe_2_-CS

Under US and N_2_ conditions, FeSe_2_ ethanol solution and BP ethanol solution were mixed in a 4:1 ratio, and stirred for 12 h in the dark. Afterward, the mixture was centrifuged at 3,000 rpm for 15 min to remove the supernatant, resuspended with excess CS solution, and stirred overnight. It was then centrifuged at 3,000 rpm for 15 min to remove the supernatant, and the precipitate was dissolved in water to obtain BP-FeSe_2_-CS (BFeSe_2_). It was stored in the dark at 4 °C.

### Stability of BFeSe_2_

First, BP, FeSe_2_, and BFeSe_2_ were dispersed in ultrapure water. Subsequently, their ultraviolet (UV)–vis absorbance was detected at days 1, 3, and 5.

### Research on the mechanism of functionalized BP heterojunction enhanced SDT

#### Electron spin resonance detection of ·OH

A total of 1 mg/ml of FeSe_2_, BP, and BFeSe_2_ solutions was taken, and the control group was set as an aqueous solution. After irradiation with 1.5 W/cm^2^ US for 3 min, electron spin resonance (ESR; Bruker E500) measurement was performed.

#### Determination of intracellular ROS and O_2_^−·^

MB49 cells were treated with FeSe_2_, BP, and BFeSe_2_ (final concentration of 50 μg/ml) for 8 h. Then, the US group was followed by US irradiation at 1.5 W/cm^2^ for 3 min. After washing twice with phosphate-buffered saline (PBS), the cells were treated with 200 μl of DCFH-DA or dihematoporphyrin ether (DHE; diluted with 1:1,000 serum-free medium) for 30 min, and then cells were washed twice with serum-free medium to remove excess DCFH-DA or DHE. The distribution of ROS or O_2_^−·^ fluorescence intensity was observed in each group using a fluorescence microscope.

#### Cyclic volt ampere curve test

1. Preparation of working electrodes

Twenty microliters of Nafion solution (which forms a polymer film to protect the electrode) was added to 500 μl of ethanol solution of FeSe_2_, BP, and BFeSe_2_ of the same concentration. US was used to thoroughly mix the two solutions at room temperature for 5 min. Then, the mixture was evenly coated on indium-tin oxide (ITO) conductive glass to form a thin film and air-dried naturally at room temperature.

2. Cyclic volt ampere curve test

Cyclic voltammetry (CV) is a commonly used electrochemical research method that involves controlling the electrode potential over time and performing one or more scans [32]. The measured substance undergoes alternating oxidation or reduction reactions on the electrode, and the current potential curve is recorded. Three-electrode systems: the prepared working electrode, the counter electrode (platinum wire), and the reference electrode (Ag/AgCl) have a voltage range of 0 to 1.0 V. The cyclic voltammogram was recorded using a PalmSens4 portable electrochemical analyzer-type electrochemical workstation.

### Cellular uptake of BFeSe_2_

The cellular uptake of BFeSe_2_ was performed according to the previous method [33]. Firstly, coumarin-6-labeled BFeSe_2_ was prepared to investigate the cellular uptake. Then, MB49 cells were seeded in a 12-well plate; lysosomes and nuclei were stained with lysotracker and 4,6-diamino-2-phenyl indole, and incubated with 10 μl of 1 mg/ml BFeSe_2_ for 0, 1, 2, 4, 8, and 12 h. The fluorescence microscope was used to observe the fluorescence.

### Antitumor effect in vitro

CCK-8: MB49 cells were seeded in 96-well plates (2 × 10^3^ cells per well) and incubated overnight. Then, FeSe_2_, BP, and BFeSe_2_ (50, 25, 12.5, 6.25, 3.125, and 0 μg/ml) were added to incubate for 24 h. After the above-mentioned treatment for 8 h, the US groups were exposed to US irradiation at 1.5 W/cm^2^ for 3 min and incubated for another 16 h. Then, the CCK-8 assay was used to determine the cell viability [34].

Calcein /PI staining: MB49 cells (4 × 10^4^ cells per well, 48-well plates) were treated with FeSe_2_, BP, and BFeSe_2_ with the same concentration, and incubated for 8 h. The US groups were exposed to US irradiation (1.5 W/cm^2^, 3 min) and incubated for another 1 h. The calcein AM/PI detection working solution was used to stain the cells. After 30 min, the cells were observed under the fluorescence microscope [35].

Flow cytometry apoptosis staining [36]: MB49 cells (2 × 10^5^ cells per dish, 6 cm dish) were treated with the same concentration of FeSe_2_, BP, and BFeSe_2_ for 8 h. The US groups were exposed to US irradiation (1.5 W/cm^2^, 3 min) and continued to be incubated for another 16 h. Then, Annexin V-APC and PI staining were performed according to the kit method, and the apoptosis cells were analyzed using flow cytometry.

Flow cell cycle detection: MB49 cells were incubated following the above method. After being treated with US irradiation and further incubated for 40 h, all of the groups were stained with PI. Finally, the flow cytometer was used to analyze the cells.

Assessment of mitochondria integrity: To evaluate the integrity of mitochondria, MB49 cells (1 × 10^4^ cells per well) were seeded in 24-well plates and incubated with the above-mentioned treatment for 8 h. For US groups, the cells were exposed to US (1.5 W/cm^2^, 3 min) and continued to be incubated for another 1 h. Last, the cells were stained with the JC-1 for 20 min and imaged on a fluorescence microscope.

Cell scratch test: MB49 cells were seeded in 6-well plates (1.2 × 10^6^ cells per well) for wound healing assay. When the cells reached a confluent state, cells were scraped by a pipette tip. The cells were incubated with the above-mentioned treatment for 8 h and the US group was followed by US irradiation at 1.5 W/cm^2^ for 3 min. The wound widths were measured at 0 and 24 h.

Colony formation assay: MB49 cells were seeded and incubated with the above-mentioned treatment in 6-well plates (2,000 cells per well) for 8 h. For US groups, the cells were exposed to US (1.5 W/cm^2^, 3 min). Then, all treatment groups were cultured for another 7 days to allow colony formation. After being washed with PBS twice, the cells were fixed with 4% polyformaldehyde for 15 min and stained with 0.5% crystal violet for 15 min at room temperature. Subsequently, they were washed with PBS twice and naturally air-dried, and the cell community coverage area was counted.

### Biological transmission electron microscopy

MB49 cells were seeded in 10-cm dishes and incubated with BFeSe_2_ (0.5 μg/ml) for 4 and 24 h, respectively. In addition, to observe the damage of different treatments on MB49 cells, a US group and a BFeSe_2_ + US group were established. Finally, the glutaraldehyde (2.5%) was used to fix the cells and biological transmission electron microscopy (Bio-TEM) was used for further observation [33].

### Untargeted metabolomics analysis

MB49 cells were seeded in 10-cm culture dishes and incubated with BFeSe_2_ (0.5 μg/ml) and US (1.5 W/cm^2^, 3 min). The cells were collected after washing twice with pre-cooled PBS. Then, the cells were dispersed into 80% methanol, treated with liquid nitrogen for 15 min, and stored at −80 °C. Finally, the Vanquish UHPLC system (Thermo Fisher) was used to perform ultra-high-performance liquid chromatography–mass spectrometry analysis on the cells [34].

### Metabolism in vivo and MRI verification:

Indocyanine green (ICG)-labeled BFeSe_2_ solution was injected into mice via the tail vein and intratumoral injection, respectively. The near-infrared fluorescence distribution of BFeSe_2_ in vivo and within the tumor was detected via the IVIS Lumina system at 0, 1, 2, 4, 8, 12, 24, and 48 h, respectively. For in vivo MRI, T2-weighted signals of BFeSe_2_ in the tumor area were collected using an MR scanner (PharmaScan70/16 US) before and after intratumoral injection [37].

### In vivo antitumor study

Briefly, 1.5 × 10^6^ MB49 cells were suspended in 100 μl of PBS and injected into the right rear back of nude mice to establish the subcutaneous tumor model. Because the tumor volume was about 120 mm^3^, we divided the mice into 8 groups (*n* = 5): G1, Control (without treatment); G2, BP; G3, FeSe_2_; G4, BFeSe_2_; G5, US; G6, BP + US; G7, FeSe_2_ + US; and G8, BFeSe_2_ + US (the dose of each group was equivalent to 10 mg/kg, the US groups were followed by US irradiation at 1.5 W/cm^2^ for 5 min). The tumor volume and body weight were monitored every 2 days. After 21 days, the blood, tumor, and major organs were collected for further assessment [38].

In addition, the MB49 orthotopic bladder tumor model could also be established according to the previously reported method [31–41]. The female nude mice were anesthetized and in supine position on a table. Then, a syringe with a plastic catheter was inserted into the bladder of the mice via the urethra. The depth was about 2 cm. After 30 s of diluted trypsin solution digestion (mixed with an equal volume of PBS) and several rinses with PBS, 50 μl of 5×10^5^ MB49-Luc cells was injected into the bladder. Finally, the syringe was removed and the mice were placed on a heating pad until it awakened. US imaging was used to confirm that the bladder tumor had been successfully established. Then, as described above, we divided the mice into 8 groups and treated them accordingly (the modified syringe was inserted into the bladder through the urethra and the corresponding drug was perfused). The bioluminescence imaging of the orthotopic bladder tumor was monitored via the D-luciferin potassium salt (15 mg/kg) and the IVIS Lumina system at days 0, 3, 6, 9, and 12. Meanwhile, the body weight of mice was recorded every 2 days. Finally, the bladder tumors of all groups were collected for H&E staining. The animal experiments involved comply with the standards of the Experimental Animal Use and Management Committee of Guangzhou Medical University.

### Statistical analysis

All data were expressed as mean ± SD. All statistical analyses were performed with GraphPad Prism software (GraphPad 8.0 for Windows) and differences of *P* < 0.05 were considered statistically significant.

### Ethics approval

All animal experiments were carried out in compliance with the guidelines and regulations set forth by the Institutional Animal Care and Use Committee at Jennio Biotech Co., Ltd. Ethics number: #JENNIO-IACUC-2023-A031.

## Results and Discussion

### Synthesis and characterization of BFeSe_2_

**Fig. 2.**
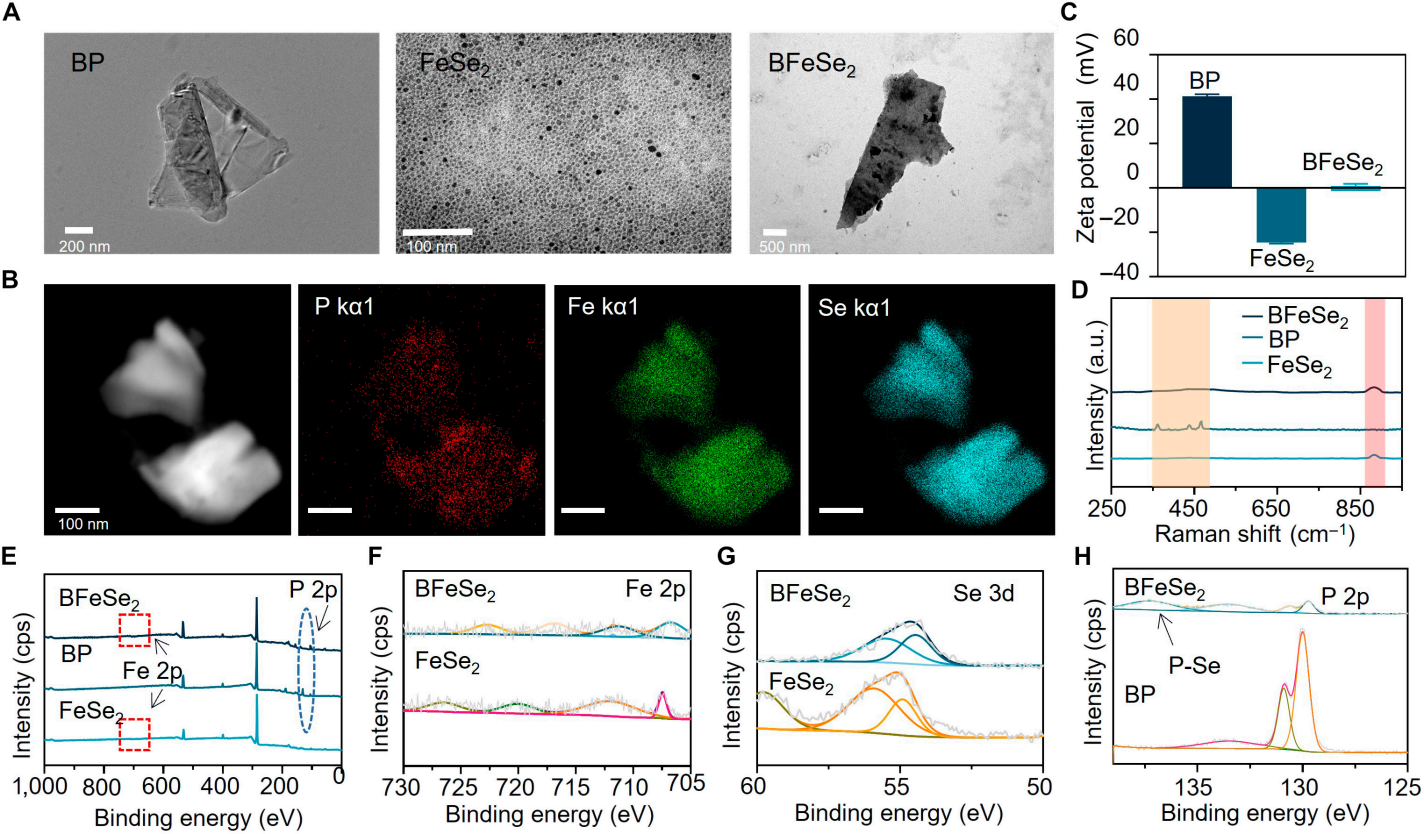
Characterization of BFeSe_2_. (A) TEM images of BP, FeSe_2_, and BFeSe_2_. (B) Corresponding elemental mapping images of BFeSe_2_. (C) Zeta potential and (D) Raman spectra of BP, FeSe_2_, and BFeSe_2_. (E) XPS spectra of FeSe_2_, BP, or BFeSe_2_. (F to H) High-resolution XPS spectra of Fe 2p, Se 3d, and P 2p_,_ and the P–Se bond was formed.

BP and FeSe_2_ were mixed in a certain proportion to form a heterojunction of BP-FeSe_2_. Then, the water solubility and bioavailability of BP-FeSe_2_ were improved by CS to further enhance its biomedical application (Fig. S1). BP-FeSe_2_ was coated with CS to obtain BP-FeSe_2_-CS composite nanoparticles (BFeSe_2_). First, the morphology of FeSe_2_, BP, and BFeSe_2_ was assessed by TEM. The BP nanosheets showed lamellar structure and the uniform size of FeSe_2_ was about 8 nm (Fig. 2A). FeSe_2_ nanoparticles were uniformly dispersed on BP. Furthermore, the elemental composition of the synthesized nanomaterials was verified by elemental mapping analysis. The results, presented in Fig. 2B and Fig. S2, highlighted the significant presence of Fe, Se, and P elements within the nanomaterials. To analyze the chemical state of BFeSe_2_, we used energy dispersive x-ray spectroscopy (EDS) and Zeta potential to analyze the elemental composition and potential changes of BFeSe_2_. In the EDS results, uniform distribution of P, Se, and Fe elements was observed in the BFeSe_2_ heterojunction (Table S1), and significant potential changes were observed in the Zeta potential when FeSe_2_ anchored onto BP (Fig. 2C). These results indicated the successful preparation of BFeSe_2_. We further measured binding energy and the chemical composition of BP, FeSe_2_, and BFeSe_2_ using XPS and Raman spectroscopy. As shown in Fig. 2D to H, FeSe_2_ bound to BFeSe_2_ by forming P–Se bonds with lone pair electrons on the surface of BP. Furthermore, to assess the impact of FeSe_2_ on BP stability, we dispersed BP, FeSe_2_, BP-FeSe_2_, and BFeSe_2_ in ultrapure water. The corresponding absorption spectra are shown in Fig. S3. The absorbance intensity of BFeSe_2_ exhibited only negligible variation, whereas the absorbance intensity of BP, FeSe_2_, and BP-FeSe_2_ decreased over time. The results showed that BFeSe_2_ had the best stability.

### The mechanism of BFeSe_2_ heterojunction enhanced SDT

**Fig. 3.**
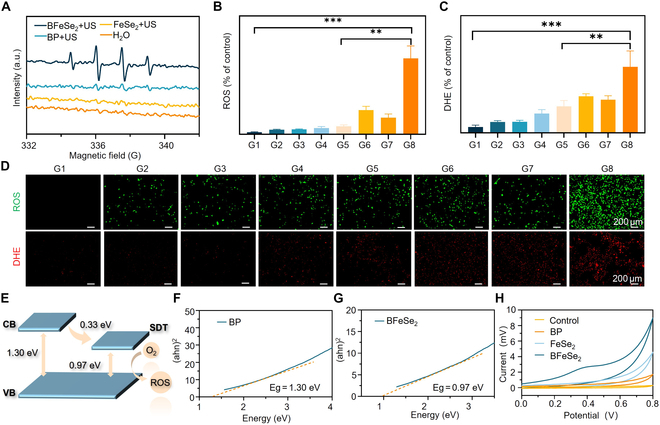
Sonodynamic mechanism of BFeSe_2_. (A) ESR spectra of •OH in H_2_O, FeSe_2_, BP, and BFeSe_2_ with US (1.5 W/cm^2^, 5 min). (B to D) The fluorescence imaging and corresponding quantitative statistical maps of total ROS and O_2_^−·^ of MB49 cells after different treatments (*n* = 3). (E) The diagram of the mechanism of BFeSe_2_ for enhanced SDT. Band gaps of (F) BP and (G) BFeSe_2_. (H) Cyclic voltammetry curves of H_2_O, FeSe_2_, BP, and BFeSe_2_. G1, Control; G2, BP; G3, FeSe_2_; G4, BFeSe_2_; G5, US; G6, BP + US; G7, FeSe_2_ + US; G8, BFeSe_2_ + US. **P < 0.01, ***P < 0.001.

Firstly, to elucidate the transfer mechanism of electron–hole pairs, we used ESR to detect the types of active radicals generated by BFeSe_2_ under irradiation. As shown in Fig. 3A, after 5 min of US irradiation, the •OH signal of BFeSe_2_ was strongest, indicating the generation of •OH radicals. Then, the intracellular production levels of ROS (green fluorescence) and O_2_^−·^ (red fluorescence) were detected using DCFH-DA and superoxide anion fluorescence (DHE) as probes, respectively. After different treatments, the green and red fluorescence signals of BFeSe_2_ + US were the strongest, indicating the highest production of ROS and O_2_^−·^ (Fig. 3B to D). Additionally, we proved that the 1:4 ratio of BP:FeSe_2_ not only had the best binding ratio [30], but also enhanced SDT more effectively. As shown in Fig. S4, when the ratio of BP:FeSe_2_ was 1:1 or 1:2, only a small fraction of FeSe_2_ was modified on the BP surface, resulting in less ROS production. However, if excessive FeSe_2_ was added (BP:FeSe_2_ ratio is 1:5), a small fraction of FeSe_2_ failed to modify the BP surface, resulting in no significant difference in the induced ROS efficiency from 1:4. Therefore, we chose the optimal ratio of BP:FeSe_2_ to be 1:4. Other ratios did not form the best binding ratio, and the efficiency of ROS production was not as good as the ratio of 1:4, which might be because the 1:4 ratio had the best electron transfer efficiency and minimized the band gap of BP. To elucidate the mechanism of enhancing the sonodynamic performance of BFeSe_2_ heterostructures, the physical properties of BP and BFeSe_2_ were studied. As shown in Fig. 3F and G, through UV-Vis-NIR diffusion spectroscopy detection and Kubelka Munk function conversion, band gaps for BP and BFeSe_2_ were computed as 1.30 and 0.97 eV, respectively. The reduction in the band gap of BFeSe_2_ helped to make it easier to be excited by the US. Then, the FeSe_2_ would efficiently collect and transport the excited electrons. Therefore, the electron–hole pairs were separated effectively, which benefited to improve the production of ROS during SDT (Fig. 3E). To further prove this conclusion, CV was measured with BFeSe_2_ modified ITO electrode in the reaction medium of sodium sulfate. As shown in Fig. 3H, compared with the BP and FeSe_2_ groups, the reduction current of BFeSe_2_ increased significantly as the potential changed from 0 to 0.8 V. This was because the electrons of BFeSe_2_ were more easily excited and transferred to the ITO electrode, while the holes remained in BFeSe_2_. BFeSe_2_ enhanced the effective transmission of electrons during electron transfer, lowering the risk of electron and hole recombination, resulting in a higher reduction current. Thus, BFeSe_2_ could enhance SDT effectively.

### In vitro cell uptake and cytotoxicity

**Fig. 4.**
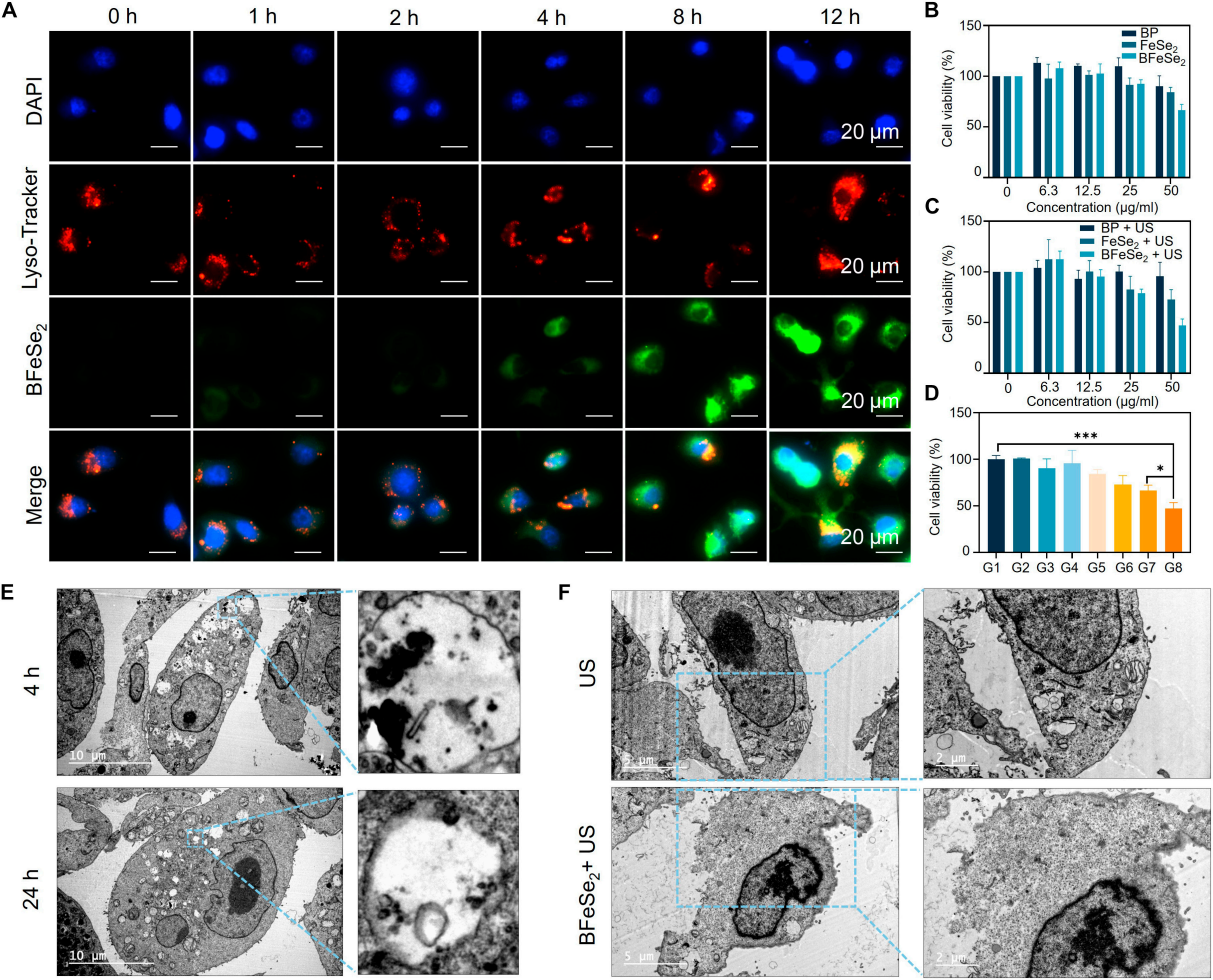
In vitro cytotoxicity and SDT efficacy. (A) Fluorescence images of the coumarin-6-labeled BFeSe_2_ in MB49 cells. (B to D) The cell viability of different treatment groups (*n* = 3). (E) Bio-TEM images of MB49 cells treated with BFeSe_2_ for 4 and 24 h. (F) Bio-TEM images of MB49 cells treated with US and BFeSe_2_ +US. G1, Control; G2, US; G3, BP; G4, BP + US; G5, FeSe_2_; G6, FeSe_2_ + US; G7, BFeSe_2_; G8, BFeSe_2_ + US. *P < 0.05, ***P < 0.001.

We first evaluated the internalization of BFeSe_2_ labeled with coumarin-6 in cancer cells. In Fig. 4A, the time-dependent uptake of cancer cells could be observed by the increasing fluorescence intensity of coumarin 6. Then, the cell viability of different treatment groups was detected using CCK-8. As shown in Fig. 4B to D, compared with the control group, the cell viability was not significantly decreased in both the US group and BFeSe_2_ group, while it significantly decreased in the BFeSe_2_ + US group (relative cell viability less than 50%). The results showed that the BFeSe_2_ + US group could significantly inhibit the cell viability of MB49 cells. BFeSe_2_ was also observed to be internalized in biological electron microscopy (Fig. 4E), and the cell structure of the BFeSe_2_ + US group was significantly disrupted (Fig. 4F). Furthermore, considering the close relationship between apoptosis and mitochondrial dysfunction, we used JC-1 dye to evaluate the mitochondrial function, which shows potential-dependent accumulation in mitochondria (red and green fluorescence indicate J-aggregates in normal mitochondrial membranes and monomers in damaged mitochondrial membranes, respectively) (Fig. S5). The results showed that except for the control group, each treatment group showed a slight increase in green fluorescence, indicating that ROS generated after different treatments could induce a decrease in mitochondrial membrane potential. An obvious green fluorescence appeared after MB49 cells were treated with the BFeSe_2_ + US group, indicating severe mitochondrial dysfunction. Moreover, the BFeSe_2_ + US group also showed the strongest red fluorescence in the live/dead cell staining test (red and green represent dead cells stained with PI and live cells stained with calcein AM, respectively), indicating the highest proportion of cell death (Fig. S6). This might be the result of the excessive increase in intracellular oxidative stress caused by BFeSe_2_-sensitized SDT-mediated production of a large amount of ROS.

To investigate the biological effects of BFeSe_2_ heterogeneous nanostructures combined with the US, we used flow cytometry to detect the proportion of MB49 cell cycle distribution. As shown in Fig. S7, the S peak in the single US group was 34.8%. However, it should be noted that the S peak in the BFeSe_2_ + US group was significantly enhanced to 51.6%. These results provided evidence for the inhibition of MB49 cell growth by BFeSe_2_ + US-induced cell apoptosis. In addition, to elucidate the anti-tumor mechanism of BFeSe_2_, we used the Annexin APC/PI dual labeling kit to analyze the apoptosis of MB49 cells under different treatments (Fig. S8). The mortality rate of the BFeSe_2_ + US group was about 29%, and the mortality rate of the US group alone and the BFeSe_2_ group were slightly higher than that of the control group. These results indicated that the cytotoxicity of the BFeSe_2_ + US group was significantly enhanced. Finally, the effects of BFeSe_2_ on the proliferation and migration of MB49 cells were validated using cloning and cell scratch tests, respectively. As shown in Fig. S9, the ability of the US group alone and the BFeSe_2_ group to inhibit the formation of MB49 cell clone communities was weaker, while the BFeSe_2_ + US group significantly inhibited the formation of MB49 cell clone communities, indicating that the proliferation of MB49 cells was effectively inhibited by the BFeSe_2_ + US group. As expected, the BFeSe_2_ + US group also inhibited the migration of MB49 cells more effectively than the other treatment groups (Fig. S10). These results demonstrated that the combined therapy significantly inhibited the proliferation and migration of MB49 cells.

### Untargeted metabolomics analysis

**Fig. 5.**
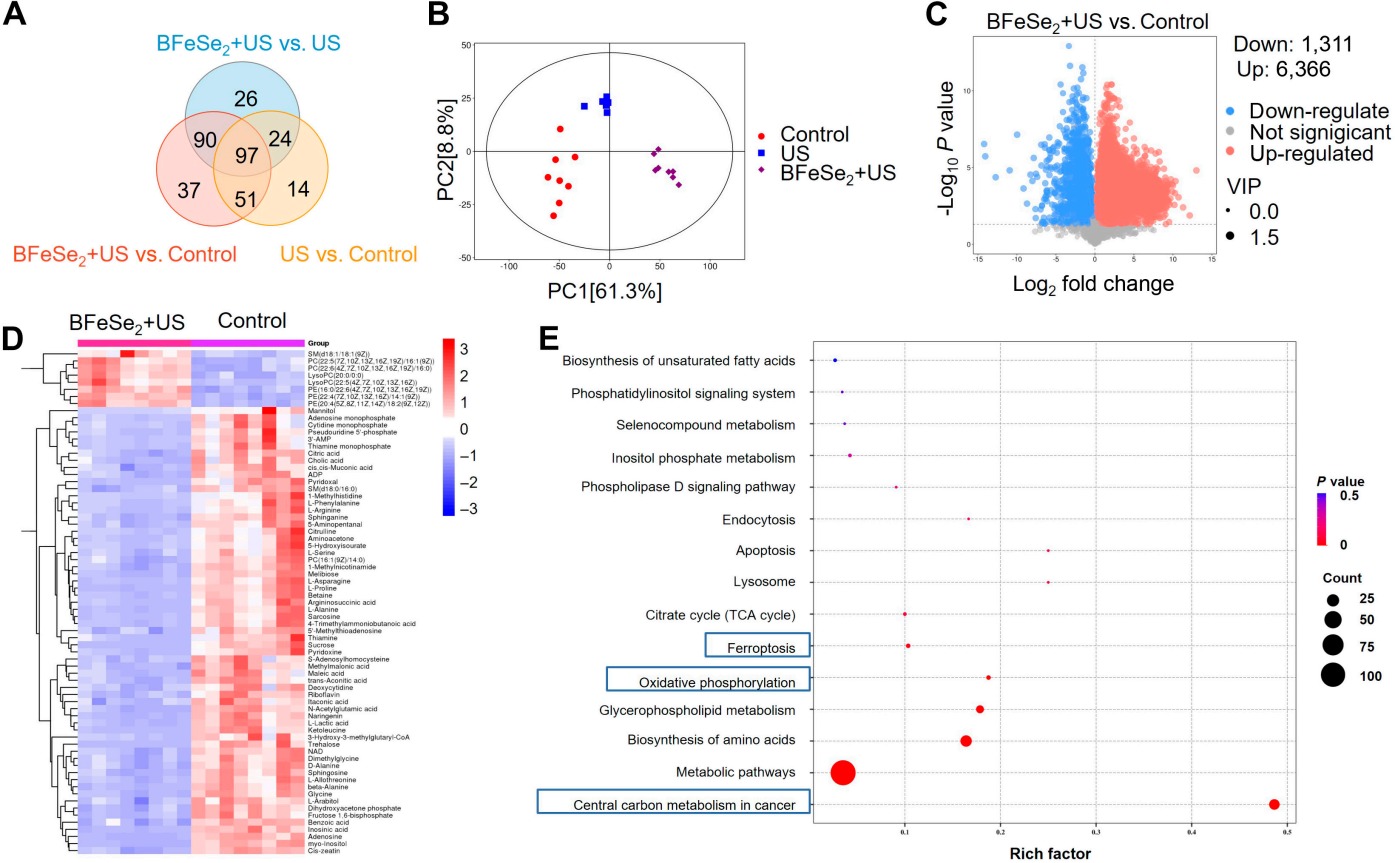
Untargeted metabolomics analysis of MB49 cells after different treatments. (A) Venn diagrams of various metabolites of different treatment groups. (B) PCA of metabolites extracted from MB49 cells with different treatments. (C) Volcanic diagrams of metabolites in MB49 cells treated with different treatments. (D) The heat maps of metabolites in MB49 cells were significantly different among different treatment groups. (E) KEGG bubble diagram of differential metabolites enrichment in pathways related to cell metabolism.

Untargeted metabolomics is a vital tool for analyzing the impacts of various treatment methods on metabolites and searching for tumor-related targets and mechanisms. Simply, we compared the various metabolites of MB49 cells treated with the control group, US group, and BFeSe_2_ + US group, respectively. As shown in Fig. 5A, the Venn diagram indicated that the metabolites of the 3 treatment groups were significantly different. The principal component analysis (PCA) scores of the BFeSe_2_ + US group were significantly separated from the control group (Fig. 5B), indicating that the difference between the 2 groups had statistical significance. The volcanic map visually displayed the overall distribution of differential metabolites (Fig. 5C). Most of the analytes were marked as gray because they were not statistically significant. There were 7,677 metabolites that were divided into up-regulation (red) and down-regulation (blue), with 6,366 metabolites up-regulated and 1,311 metabolites down-regulated, showing significant differences. Then, according to the corresponding peak area, we compared the relative levels of metabolites between the control group and the BFeSe_2_ + US group by a heat map analysis (Fig. 5D). Further investigation by Kyoto Encyclopedia of Genes and Genomes (KEGG) signaling pathway enrichment analysis of these metabolites revealed that they clearly participated in central carbon metabolism in cancer, oxidative phosphorylation, and ferroptosis (Fig. 5E). Additionally, as shown in Fig. S11A, the distribution of differential metabolites had significant differences (6,052 metabolites up-regulated and 940 metabolites down-regulated) between the BFeSe_2_ + US group and the US group. The KEGG signaling pathway enrichment analysis of 2 groups’ metabolites revealed that the they also clearly participated in the regulation of central carbon metabolism in cancer (Fig. S11B). These results suggested that BFeSe_2_ + US therapy significantly regulated the metabolic level, effectively blocked tumor-related pathways, and ultimately inhibited tumor proliferation.

### In vivo biological distribution and imaging function

To investigate the biological distribution of BFeSe_2_ in vivo, ICG-labeled BFeSe_2_ was injected into the tail vein of tumor-bearing nude mice and the fluorescence distribution in vivo was monitored. The experimental results showed that fluorescence appeared first in the liver after administration and reached its maximum value within 4 h. Subsequently, liver fluorescence weakened, and tumor tissue began to show fluorescence, which completely disappeared within 24 h (Fig. S12). This indicated that BFeSe_2_ first accumulated in the liver after entering the blood system, and then rapidly metabolized through organs such as the kidneys. We further observed the retention of BFeSe_2_ in the tumor by injecting ICG-labeled BFeSe_2_ into the tumor. Figure S13A and B showed that in the tumor area of nude mice, the fluorescence signal gradually increased, reaching its peak at the first hour. After 24 h of continuous observation, significant fluorescence signal was still observed in the tumor area. Subsequently, fluorescence imaging was performed on the main organs and tumor tissue, and fluorescence was only found in the tumor tissue (Fig. S13C). The results indicated that BFeSe_2_ could remain in the tumor area for a long time after intratumoral injection, without causing enrichment of major organs, and has good biological safety, which is beneficial for subsequent imaging and treatment. To verify the effectiveness of BFeSe_2_ in enhancing the T2 signal, MRI was used to capture the images of tumor regions in nude mice before and after intratumoral injection. The results showed that the tumor area after injection was significantly darker than before, which was consistent with the change in the value of T2, indicating that BFeSe_2_ had the effect of enhancing the T2 signal (Fig. S14).

### In vivo antitumor study

**Fig. 6.**
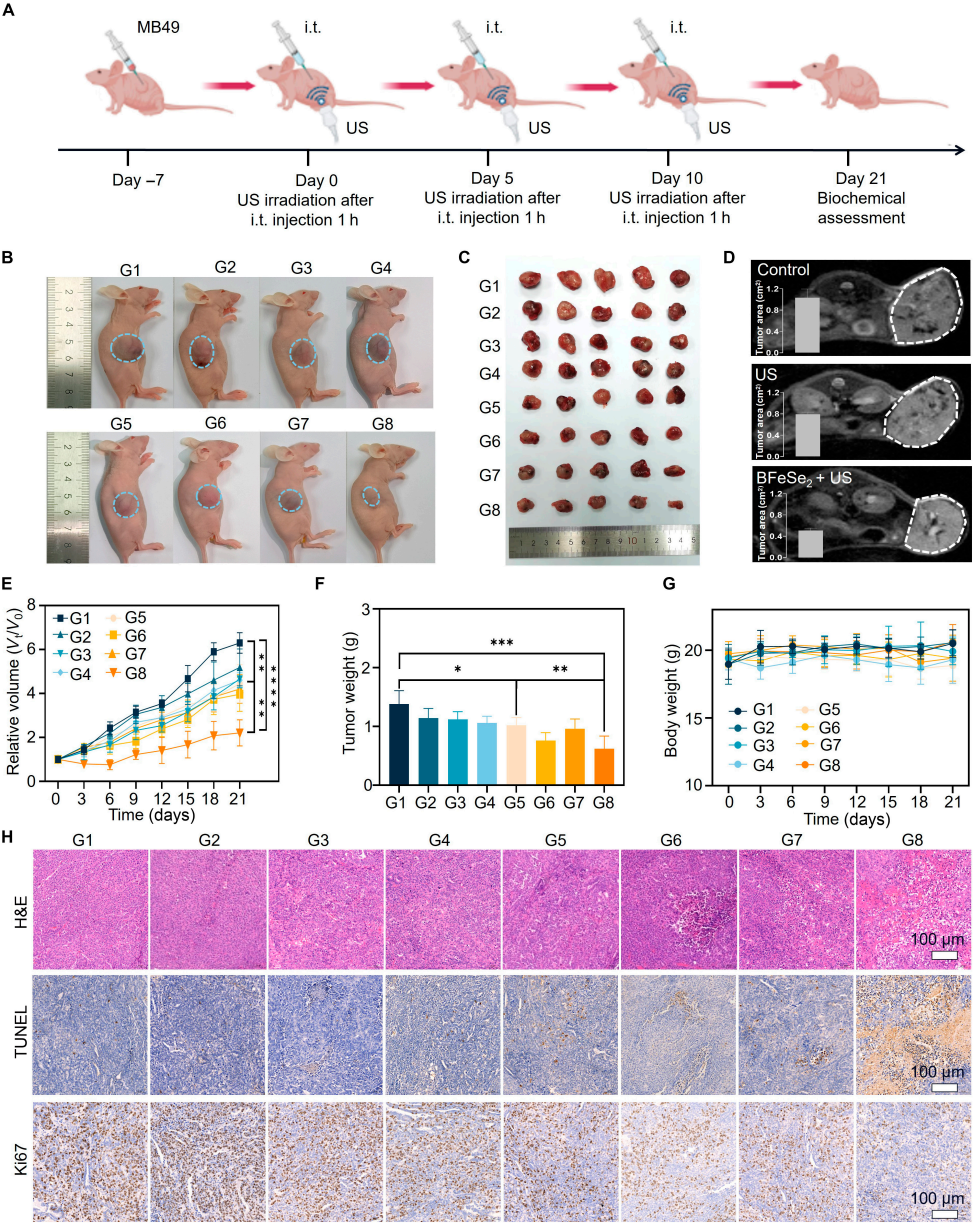
The antitumor effect in the MB49 subcutaneous tumor model. (A) Schematic diagram of the treatment plan of BFeSe_2_ in MB49 tumor-bearing mice. The photograph of (B) mice and (C) tumor, (D) MRI images and tumor area statistical charts, (E) tumor relative volume, (F) tumor weight, and (G) body weight of the MB49 tumor-bearing model in each group. (H) Representative images of H&E staining, TUNEL histochemical, and Ki-67 immune histochemical. G1, Control; G2, BP; G3, FeSe_2_; G4, BFeSe_2_; G5, US; G6, BP + US; G7, FeSe_2_ + US; G8, BFeSe_2_ + US. *P < 0.05, **P < 0.01, ***P < 0.001, ****P < 0.0001.

Based on the excellent intracellular SDT of BFeSe_2_, we further evaluated the anti-tumor effect of BFeSe_2_ in the MB49 subcutaneous tumor model using the experimental program shown in Fig. 6A. US irradiation (1.5 W/cm^2^, 5 min) was performed at the time point of maximum accumulation of BFeSe_2_. Then, tumor growth was monitored every 2 days for 21 days. After 21 days of treatment, the changes in tumor volume of mice in each group were recorded in Fig. 6B, which showed a fair therapeutic effect of the BFeSe_2_ + US group. Similarly, as shown in Fig. 6C, the excellent therapeutic effect of the BFeSe_2_ + US group was also clearly shown by the image of the excised tumor at the end of treatment compared to the other groups. The MRI images and tumor area statistical charts in Fig. 6D showed that the BFeSe_2_ + US group had the smallest tumor area, which also intuitively confirmed its tumor suppression effect. The changes observed in the relative tumor volume and weight demonstrated that the BFeSe_2_ + US group exhibited remarkable antitumor effects by effectively inhibiting bladder tumor growth in MB49 (Fig. 6E and F). There was no remarkable change in the body weight of mice between different groups over the treatment period, indicating the low systemic toxicity of the BFeSe_2_ (Fig. 6G). In addition, to study the inhibitory mechanism of BFeSe_2_ on bladder cancer in vivo, the treated tumor tissue sections were used for H&E, Ki67, and TUNEL staining (Fig. 6H). The BFeSe_2_ + US group exhibited severe histological damage, much less proliferation, and high cell apoptosis, all of which were the reasons for tumor growth inhibition. The safety of BFeSe_2_ was further verified by blood biochemical tests and H&E observations of major organs at the end of treatment, exhibiting no acute cytotoxicity and significant tissue damage (Figs. S15 and S16).

**Fig. 7.**
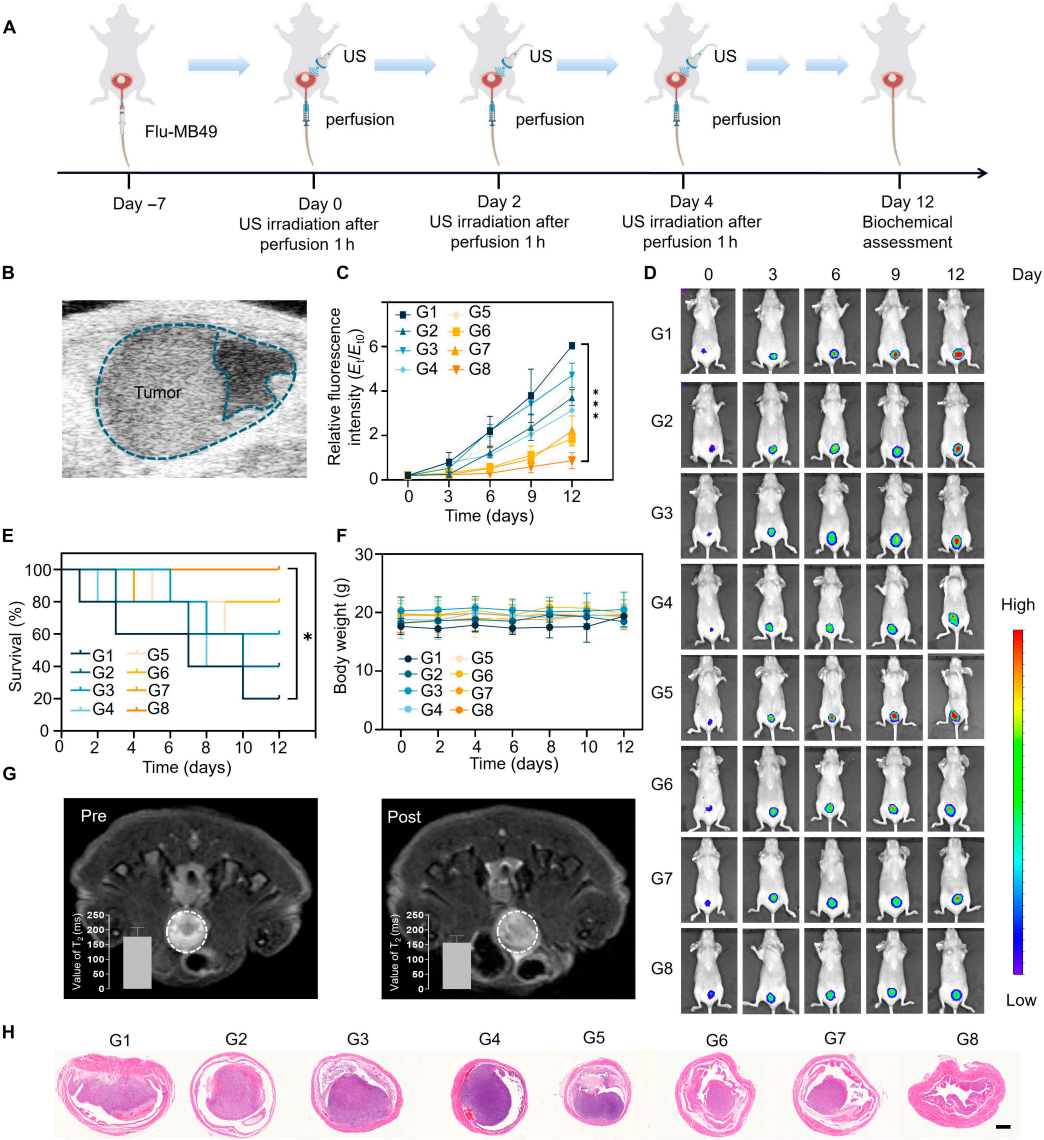
The antitumor effect in the MB49 orthotopic bladder cancer model. (A) Schematic diagram of the treatment plan of BFeSe_2_ in the MB49 orthotopic bladder cancer model. (B) The ultrasound images of the orthotopic bladder tumor. (C) Relative fluorescence intensity, (D) in vivo bioluminescence images, (E) survival rate, and (F) body weight of MB49 orthotopic cancer model in each group. (G) MRI images and the intensity of MRI signals of the MB49 orthotopic bladder cancer model before and after perfusion of BFeSe_2_. (H) Representative images of H&E staining of the bladder tumors (scale bar, 500 μm). G1, Control; G2, BP; G3, FeSe_2_; G4, BFeSe_2_; G5, US; G6, BP + US; G7, FeSe_2_ + US; G8, BFeSe_2_ + US. *P < 0.05, ***P < 0.001.

In addition, we also studied the anti-tumor therapeutic effect of BFeSe_2_ by establishing an MB49 orthotopic bladder tumor model. Briefly, as shown in Fig. 7A, we injected MB49-Luc cells (which have the property of bioluminescence shown in Fig. S17) into the bladder. After 1 week, the US imaging had shown that there was tumor tissue in the bladder (Fig. 7B), indicating that the MB49 orthotopic bladder tumor model had been successfully established [39]. Subsequently, we used the IVIS Lumina system to obtain bioluminescence imaging (recorded as day 0). Then, we randomly divided the mice into 8 groups and intravesically perfused them 3 times with different drugs (2 days/time). Bioluminescence imaging was recorded on days 0, 3, 6, 9, and 12. The results showed that among all treatment groups, the BFeSe_2_ + US group had the best antitumor efficacy, consistent with the relative fluorescence intensity (Fig. 7C and D). Furthermore, the survival situation of mice was also monitored and recorded during the treatment. The survival rate of the control group was only 20%, while that of the BFeSe_2_ + US group was 100% (Fig. 7E), indicating that the BFeSe_2_ + US group exhibited significant antitumor ability. The excised bladder of the BFeSe_2_ + US group was significantly smaller than that of the other treatment groups (Fig. S18). Over a 12-day treatment period, there was no obvious change in the body weight of each group of mice (Fig. 7F). In addition, to further validate the effect of BFeSe_2_ on enhancing the T2 signal in the bladder tumor region, the T2-weighted MRI images before and after perfusion of BFeSe_2_ were compared. As shown in Fig. 7G, the values of T2 after perfusion in the tumor region were lower than before, which were consistent with the darkening effects of the images, indicating that BFeSe_2_ had the effect of enhancing the T2 signal. For H&E-stained samples, compared to other intravesical administration, when mice received treatment with the BFeSe_2_ + US group, the bladder tumor significantly shrank (Fig. 7H), which was consistent with the results of bioluminescence imaging. In summary, BFeSe_2_ combined with US in the treatment of bladder in situ tumors had a significant inhibitory effect on tumor growth and could enhance the T2 signal in the tumor area.

## Conclusion

In summary, we had prepared FeSe_2_ anchored BP through P–Se bonding as a BP heterojunction sonosensitizer (BFeSe_2_). By adjusting the compositional ratio of BP to FeSe_2_, BFeSe_2_ had the ability to efficiently induce ROS production under US. The FeSe_2_ could capture the excited electrons and reduce the band gap of BP, thus improving the separation of electron–hole pairs between FeSe_2_ and BP could enhance the sonodynamic effect. BFeSe_2_ had high stability, high biocompatibility, and high sonodynamic efficiency, could enhance the SDT of bladder cancer, effectively inhibited tumor growth, and had low systemic toxicity on the nude mice. Moreover, the abundant Fe^2+^ in BFeSe_2_ could effectively improve the T2 signal for tumor-specific MRI. Therefore, there is great potential for BFeSe_2_ in the field of bladder cancer diagnosis and treatment.

## Data Availability

The datasets used and/or analyzed during the current study are available from the corresponding author on reasonable request.
