# Supplementary material for: Ferrous Selenide Stabilized Black Phosphorus Heterojunction Sonosensitizer for MR Imaging-Guided Sonodynamic Therapy of Bladder Cancer
Source: Biomater Res. 2024 Mar 27;28:0014. doi: 10.34133/bmr.0014 (PMC10976587; doi:10.34133/bmr.0014)
Supplement: Supplementary 1 — Table S1 Figs. S1 to S18 [file bmr.0014.f1.docx]

**Supporting Information**

**Ferrous selenide stabilized black phosphorus heterojunction sonosensitizer for MR imaging****-guided sonodynamic therapy of bladder cancer**

Sicheng Wu^1^†, Guanlin Li^1^†, Wenrui Ouyang^1^†, Yuan Tian^1^, Shujue Li^1^, Wenqi Wu^2^, Hongxing Liu^1^*

1. Guangdong Provincial Key Laboratory of Urology, Guangdong Engineering Research Center of Urinary Minimally invasive surgery Robot and Intelligent Equipment, Guangzhou Institute of Urology, Department of Urology, The First Affiliated Hospital of Guangzhou Medical University, Guangzhou Medical University, Guangzhou, 510120, China.

2. The Second Affiliated Hospital of Guangzhou Medical University, Guangzhou Medical University, Guangzhou, China

* Corresponding authors:

Hongxing Liu, [liuhongxing@gzhmu.edu.cn](mailto:liuhongxing@gzhmu.edu.cn);

† Sicheng Wu, Guanlin Li, and Wenrui Ouyang contributed equally to this work.

**Table S1**. The EDX content proportion of BFeSe_2_.


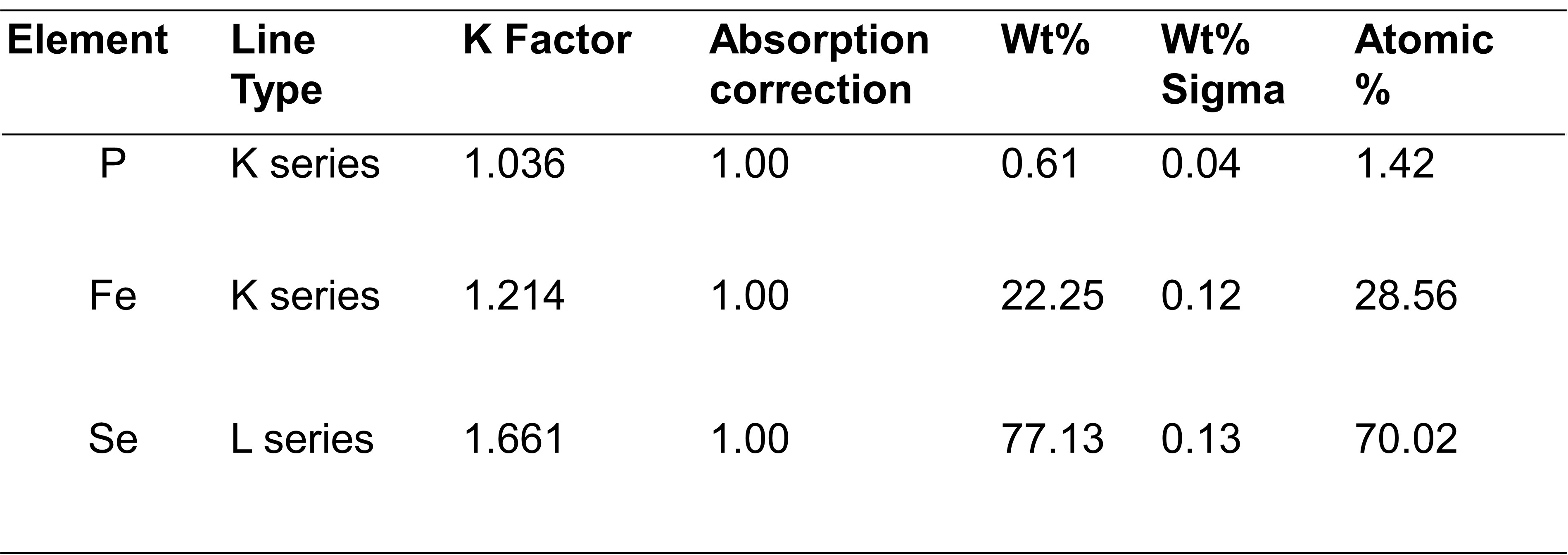


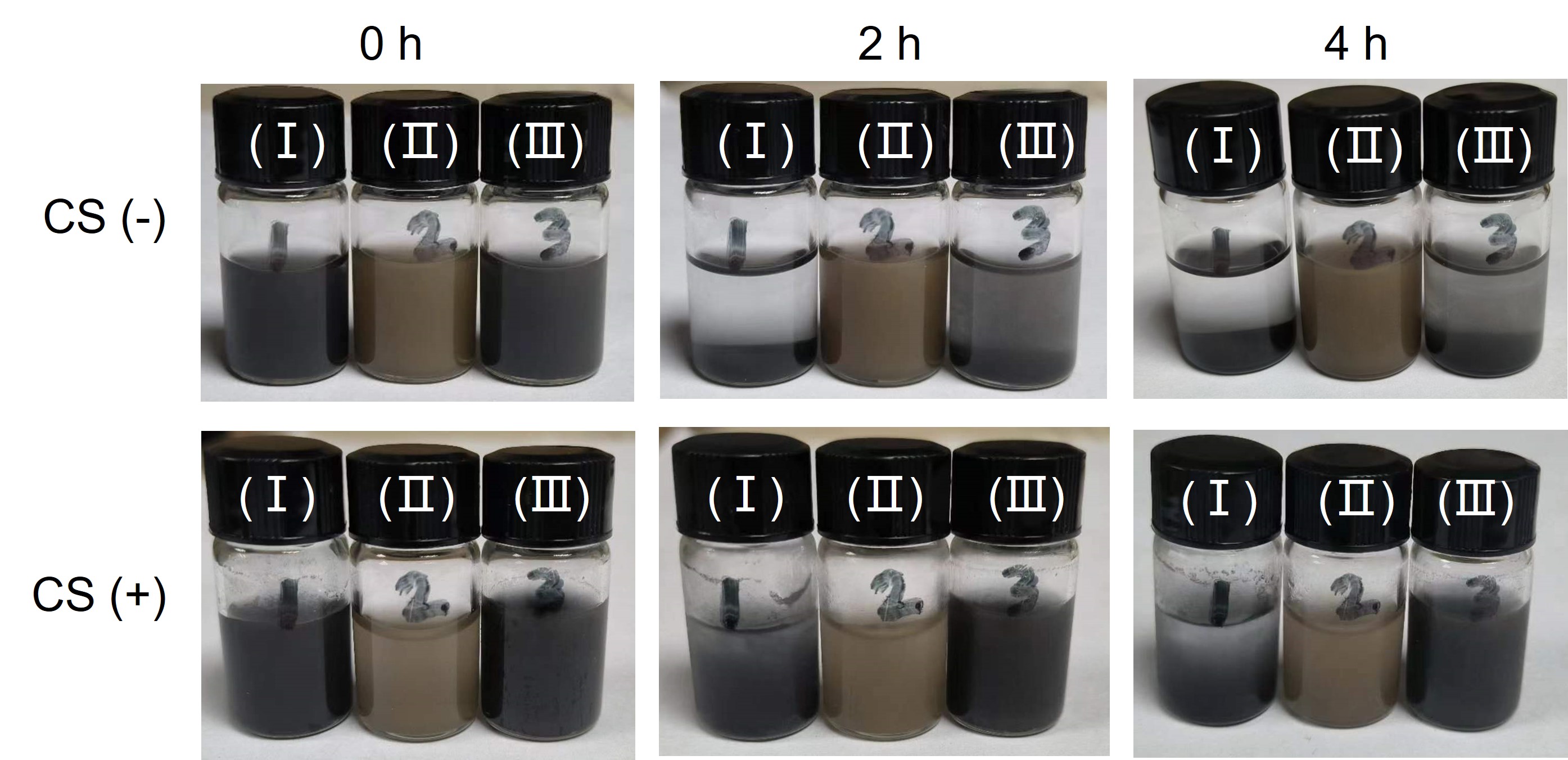


**Figure S1.** Photographs of FeSe_2_, BP and BP-FeSe_2_ decorated with/without CS. (Ⅰ：FeSe_2_; Ⅱ：BP; Ⅲ: BP-FeSe_2_).


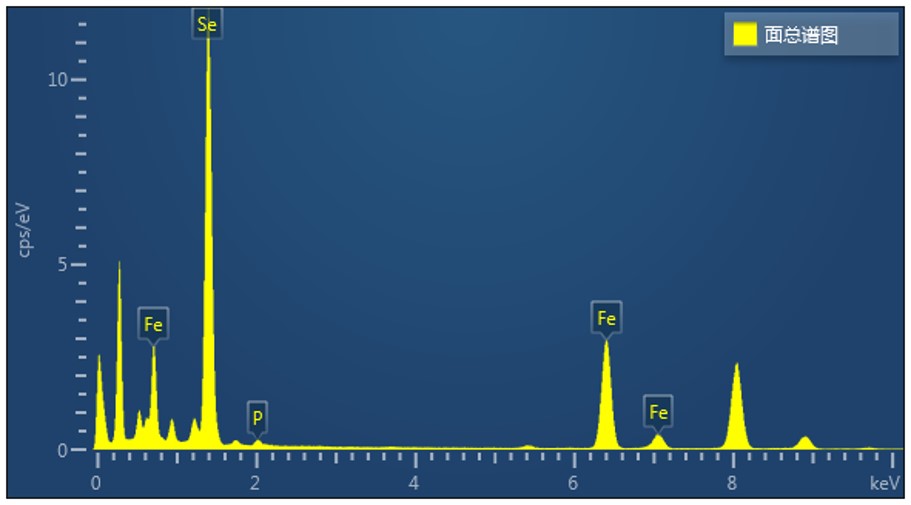


**Figure S2**. The EDX spectrum of BFeSe_2_.


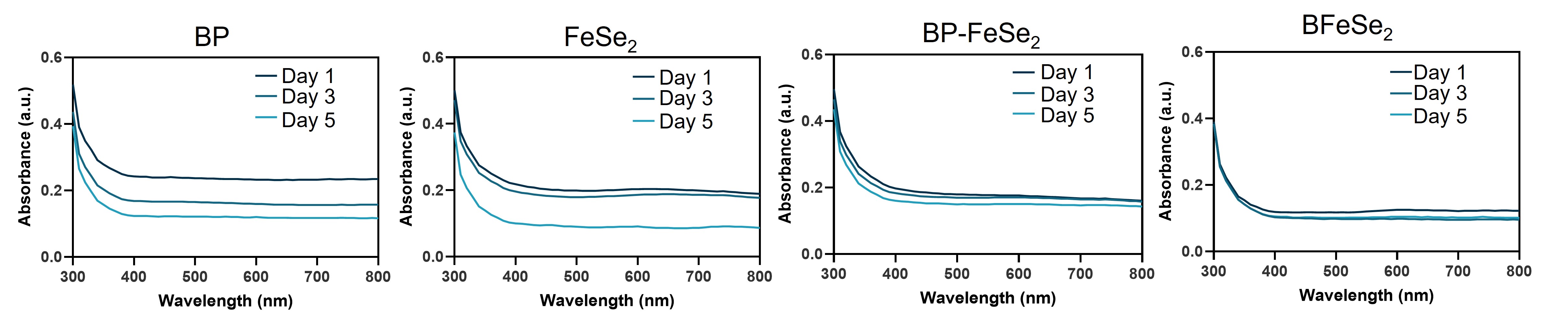


**Figure S3.** 5-day UV-vis absorption spectra of BP, FeSe_2,_ BPs-FeSe_2_ and BFeSe_2_ solutions.


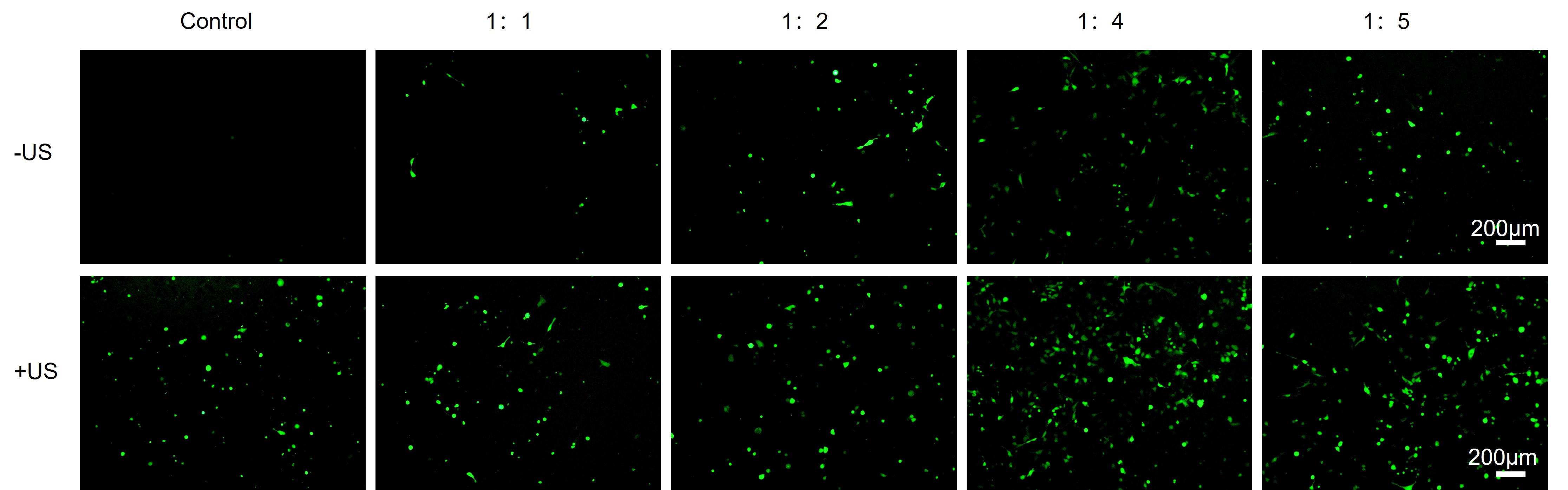


**Figure S4.** ROS condition of MB49 cells treated with BFeSe_2_ (BP: FeSe_2_ = 1:1, 1:2, 1:4, 1:5) with/without US irradiation.


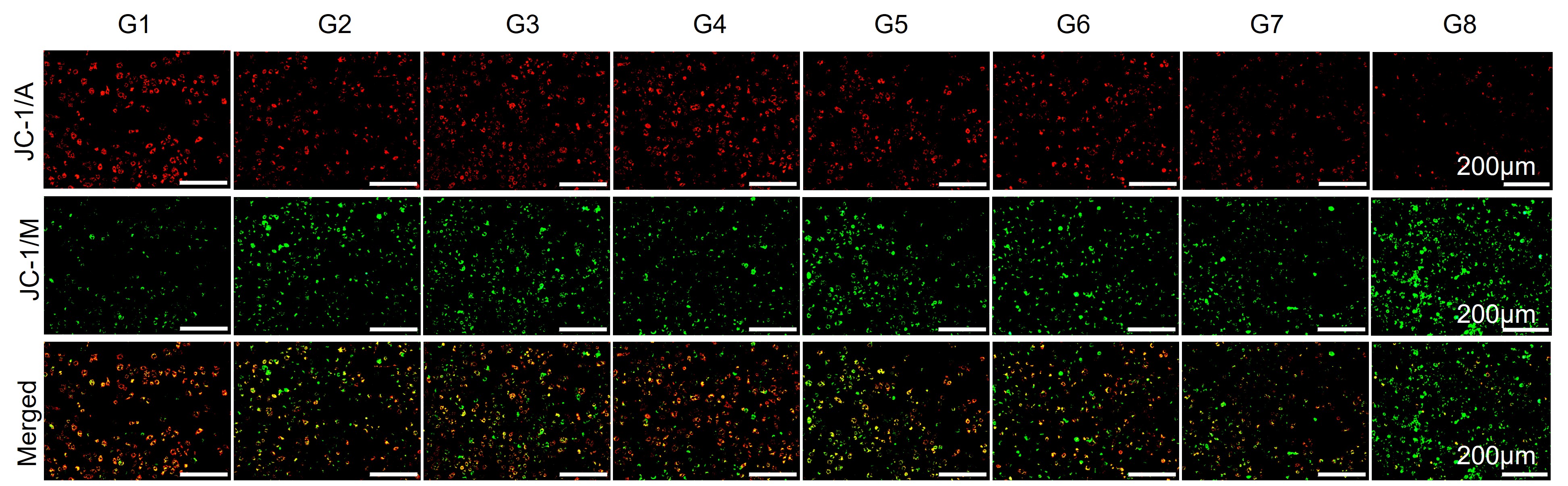


**Figure S5.** Images of MB49 cells stained with JC-1 after different treatments. (G1. Control, G2. BP, G3. FeSe_2_, G4. BFeSe_2_, G5.US, G6. BP + US, G7. FeSe_2_ + US, G8. BFeSe_2_ + US).


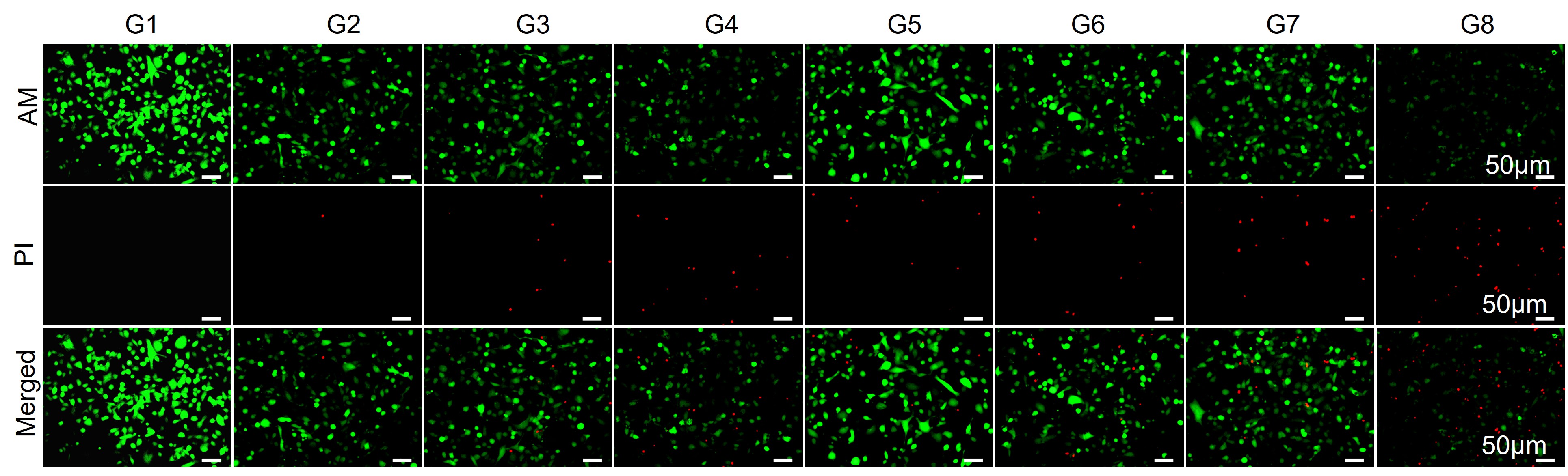


**Figure S6.** Live/dead cell staining to visualize the cell viability of MB49 cells under different treatments. (G1. Control, G2. BP, G3. FeSe_2_, G4. BFeSe_2_, G5.US, G6. BP + US, G7. FeSe_2_ + US, G8. BFeSe_2_ + US).


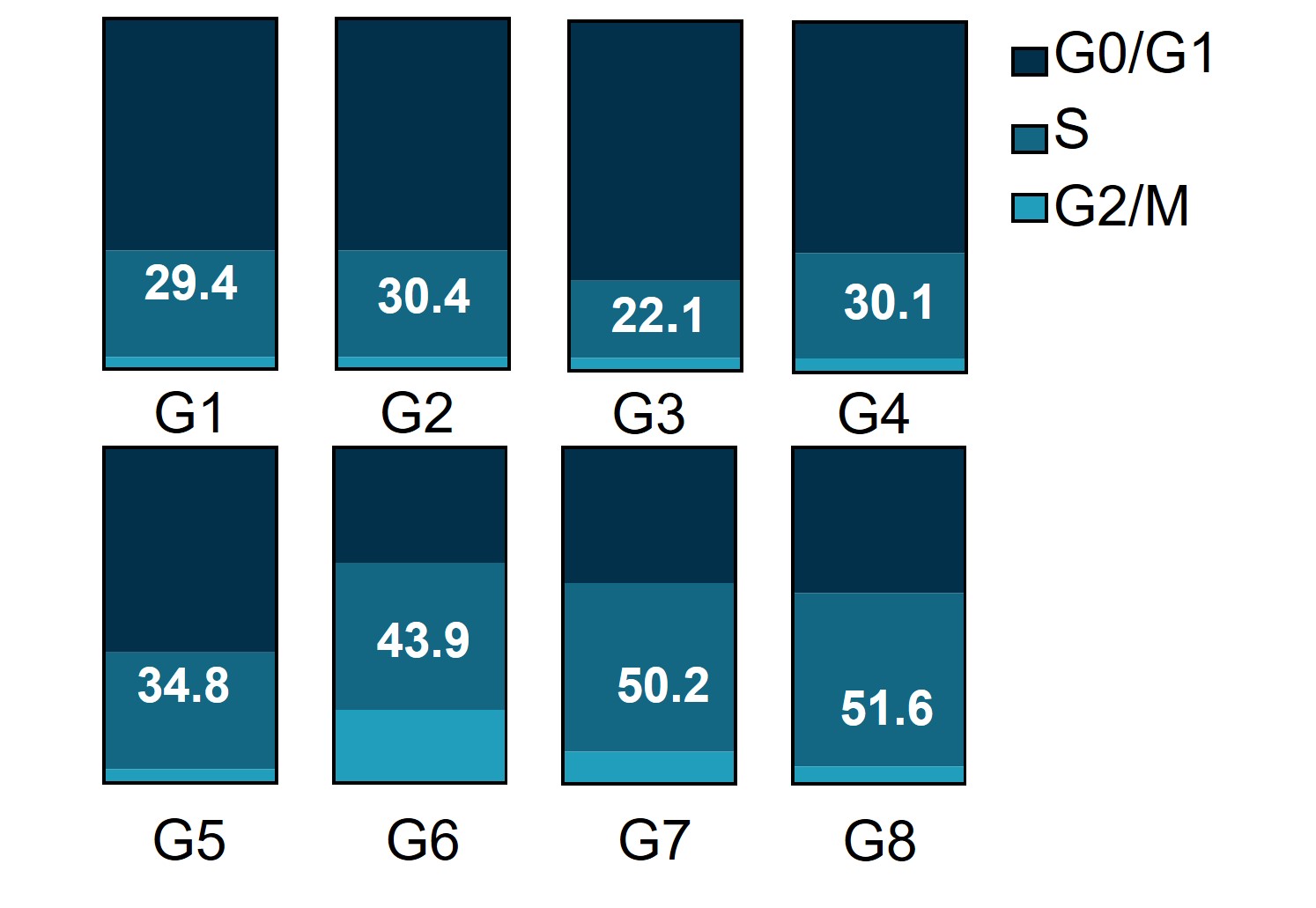


**Figure S7.** Histogram of cell cycle phase of MB49 cells under different treatments. (G1. Control, G2. BP, G3. FeSe_2_, G4. BFeSe_2_, G5.US, G6. BP + US, G7. FeSe_2_ + US, G8. BFeSe_2_ + US).


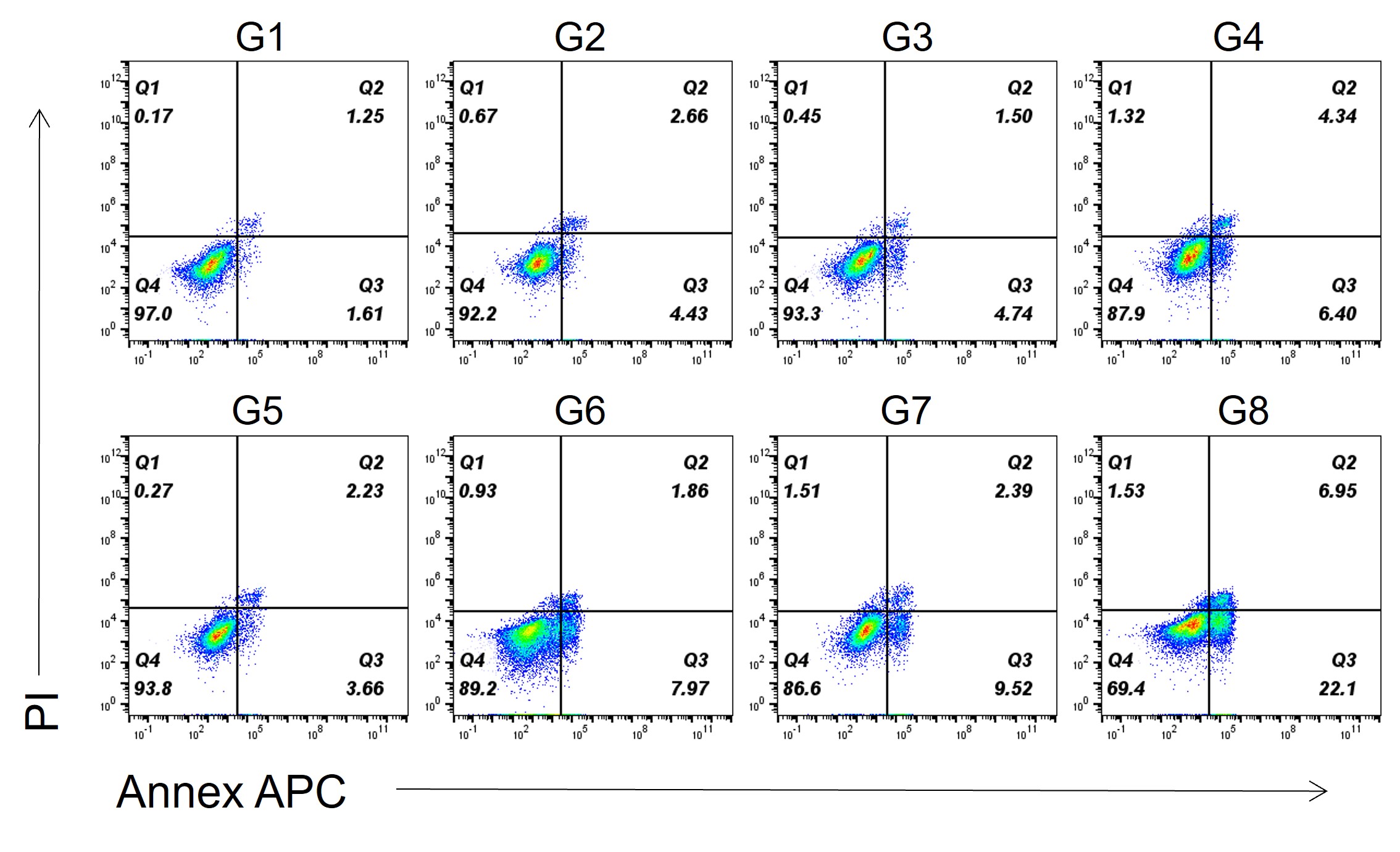


**Figure S8.** Flow cytometry analysis of MB49 cell apoptosis using the Annexin APC and PI staining. (G1. Control, G2. BP, G3. FeSe_2_, G4. BFeSe_2_, G5.US, G6. BP + US, G7. FeSe_2_ + US, G8. BFeSe_2_ + US).


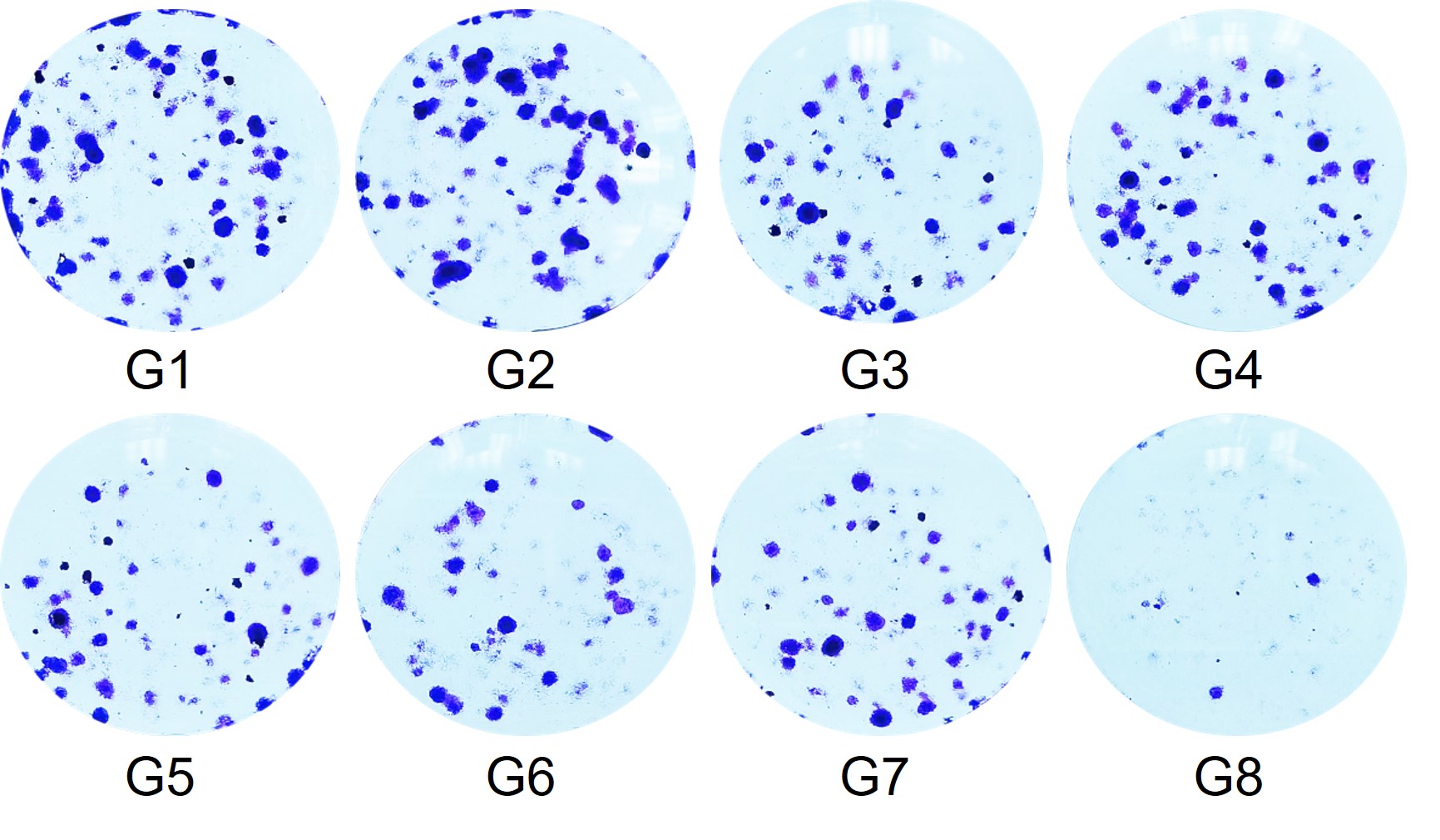


**Figure S9.** The colony formation of MB49 cells treated with different treatments. (G1. Control, G2. BP, G3. FeSe_2_, G4. BFeSe_2_, G5.US, G6. BP + US, G7. FeSe_2_ + US, G8. BFeSe_2_ + US).


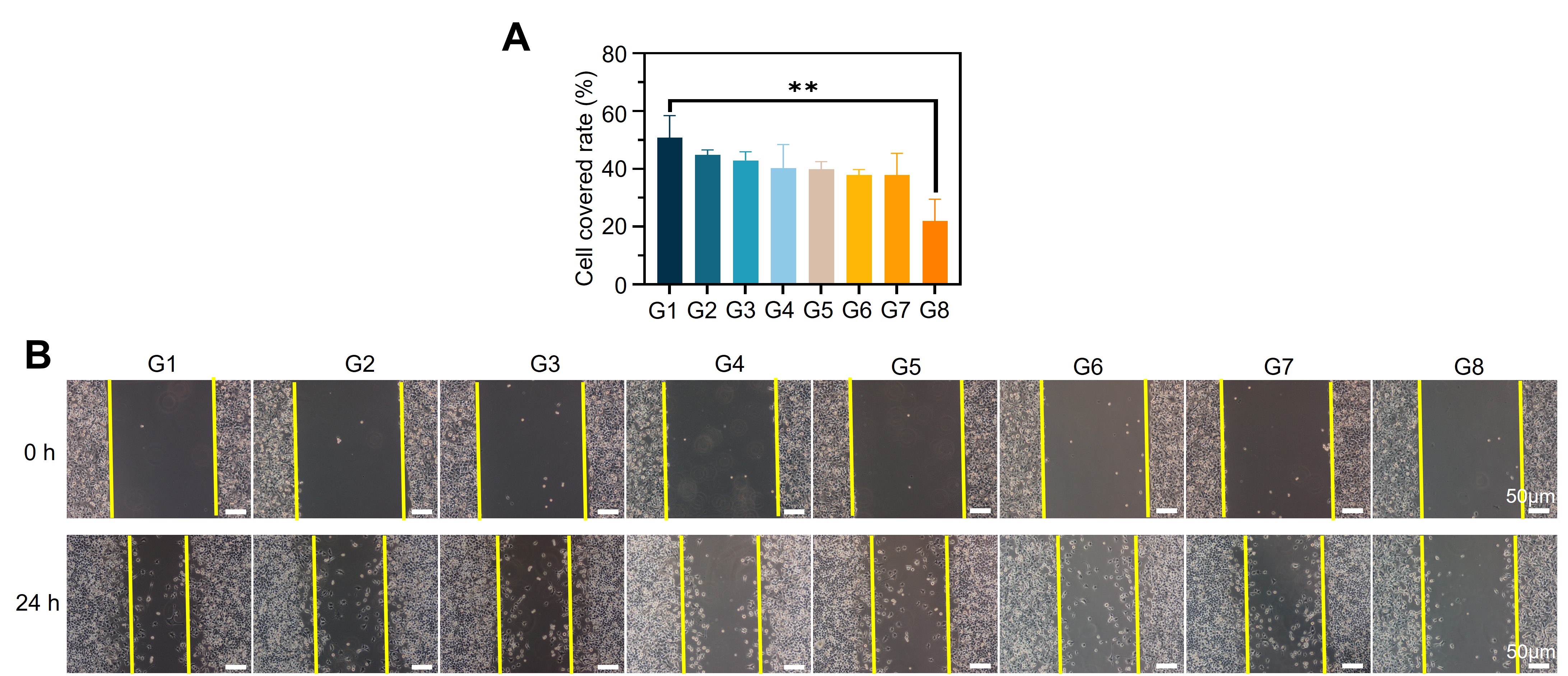


**Figure S10.** Wound healing assay analysis. (A) The cell covered rate and (B) photos of MB49 cells after different treatments. (G1. Control, G2. BP, G3. FeSe_2_, G4. BFeSe_2_, G5.US, G6. BP + US, G7. FeSe_2_ + US, G8. BFeSe_2_ + US). (**P < 0.01).


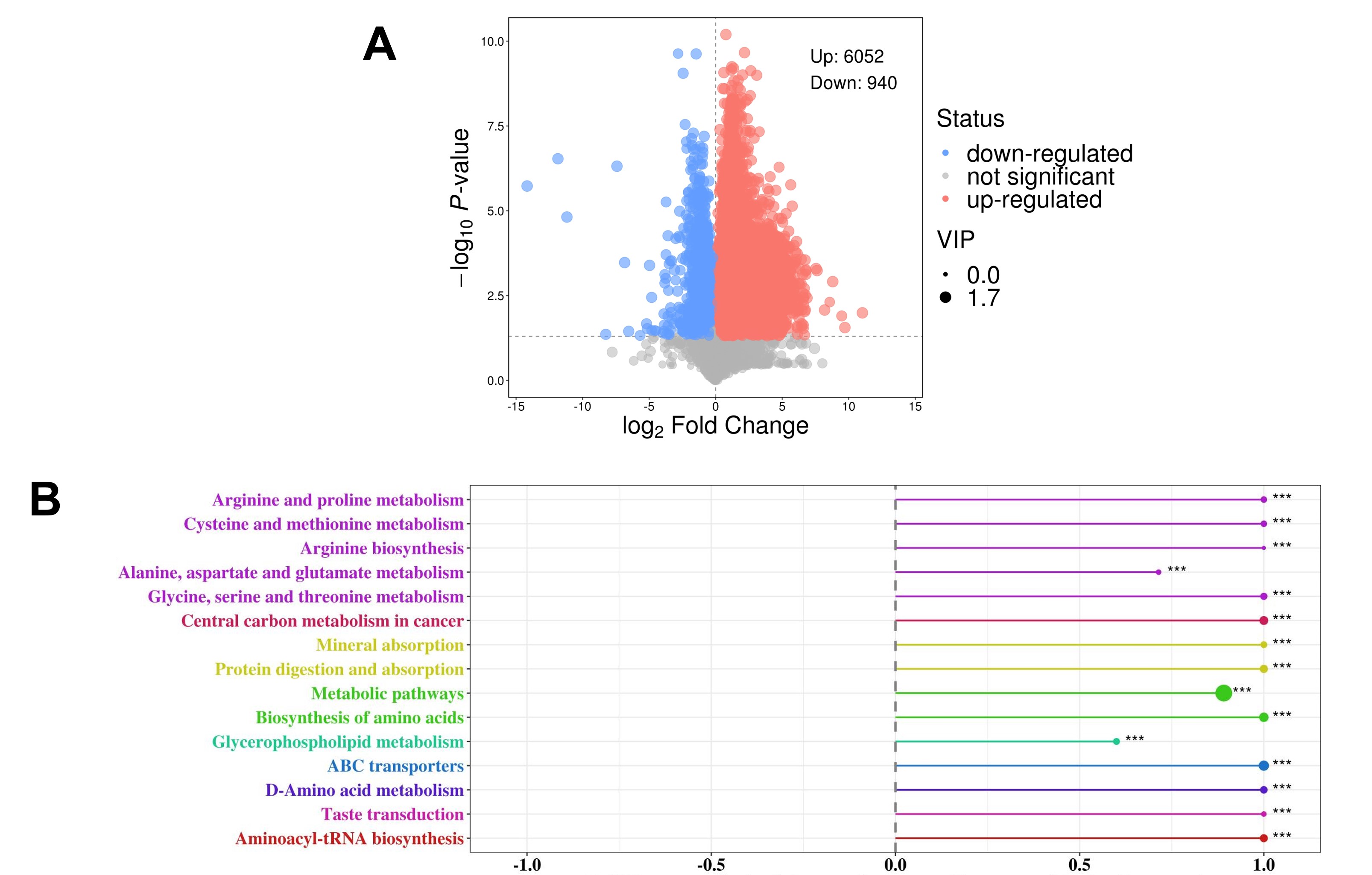


**Figure S11.** Untargeted metabolomics analysis of MB49 cells after treat with BFeSe_2_ + US and US. (A) Volcanic diagrams of metabolites in MB49 cells treated with BFeSe_2_ + US group and the US group. (B) KEGG DA Score plot of differential metabolites enrichment in pathways related to cell metabolism.

**
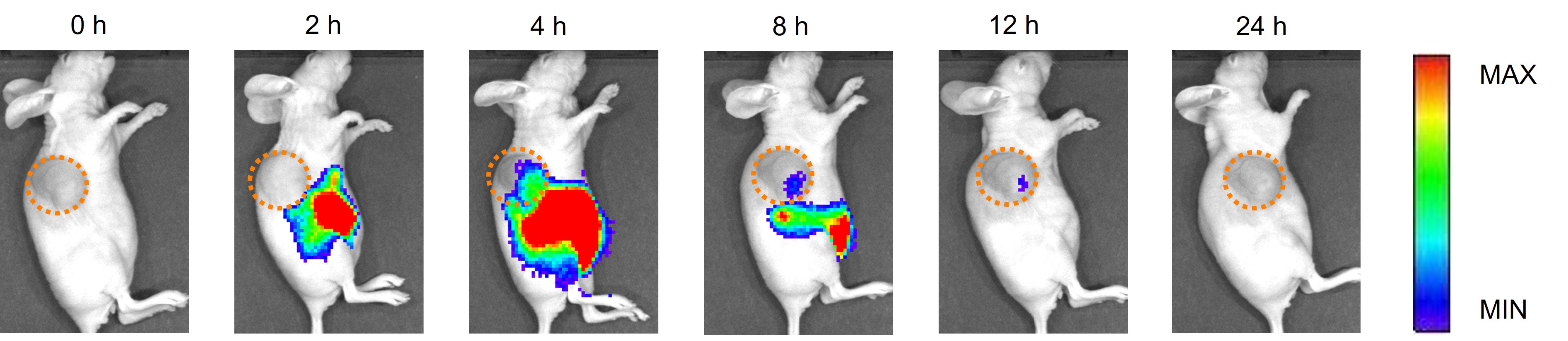
Figure S12.** Time-dependent in *vivo* fluorescence imaging of MB49 tumor-bearing mice post i.v. injection of ICG-labeled BFeSe_2_. The circles indicate the tumor region.

**
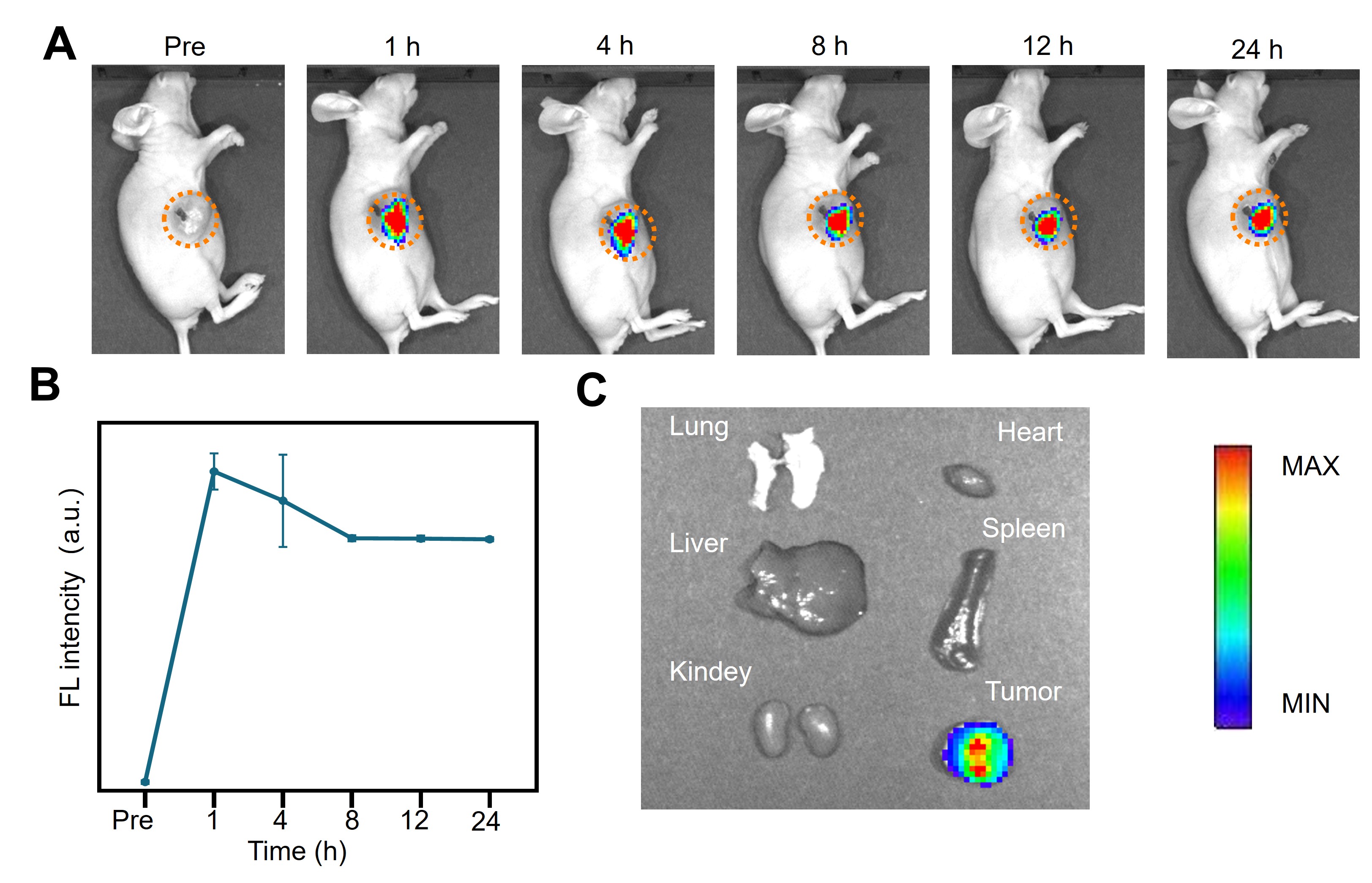
Figure S13.** In *vivo* biodistribution. (A) Time-dependent in *vivo* fluorescence imaging and (B) quantification of MB49 tumor-bearing mice post i.t. injection of ICG-labeled BFeSe_2_. The circles indicate the tumor region. (C) The fluorescence imaging of tumor and main organs.

**
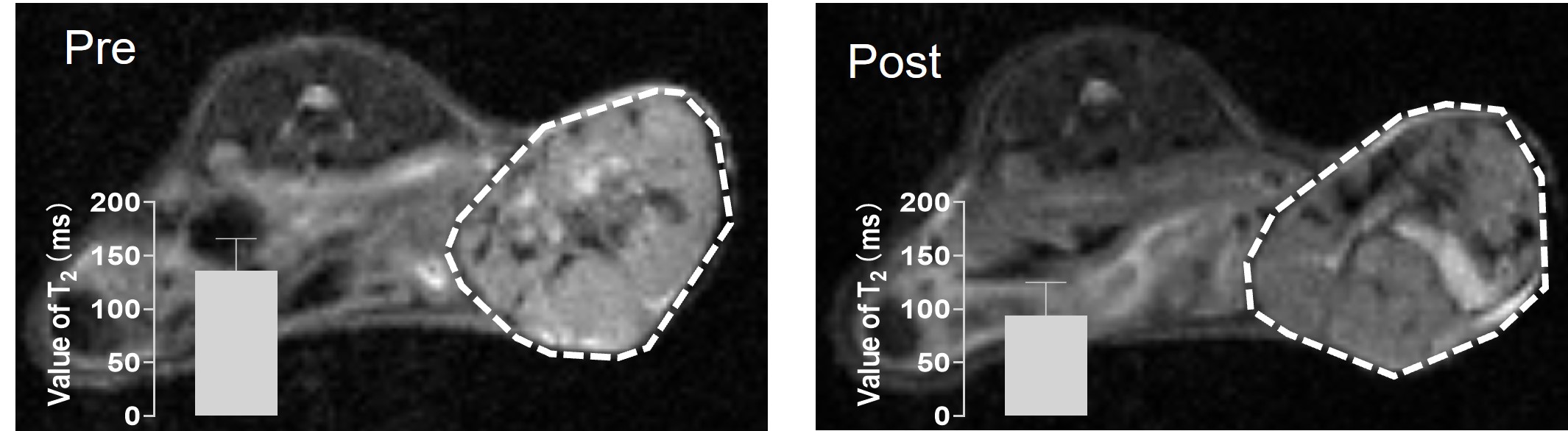
Figure S14.** In vivo MR imaging and the intensity of MRI signals of MB49 tumor-bearing mice before and after i.t. injection of BFeSe_2_ at 1 h.

**
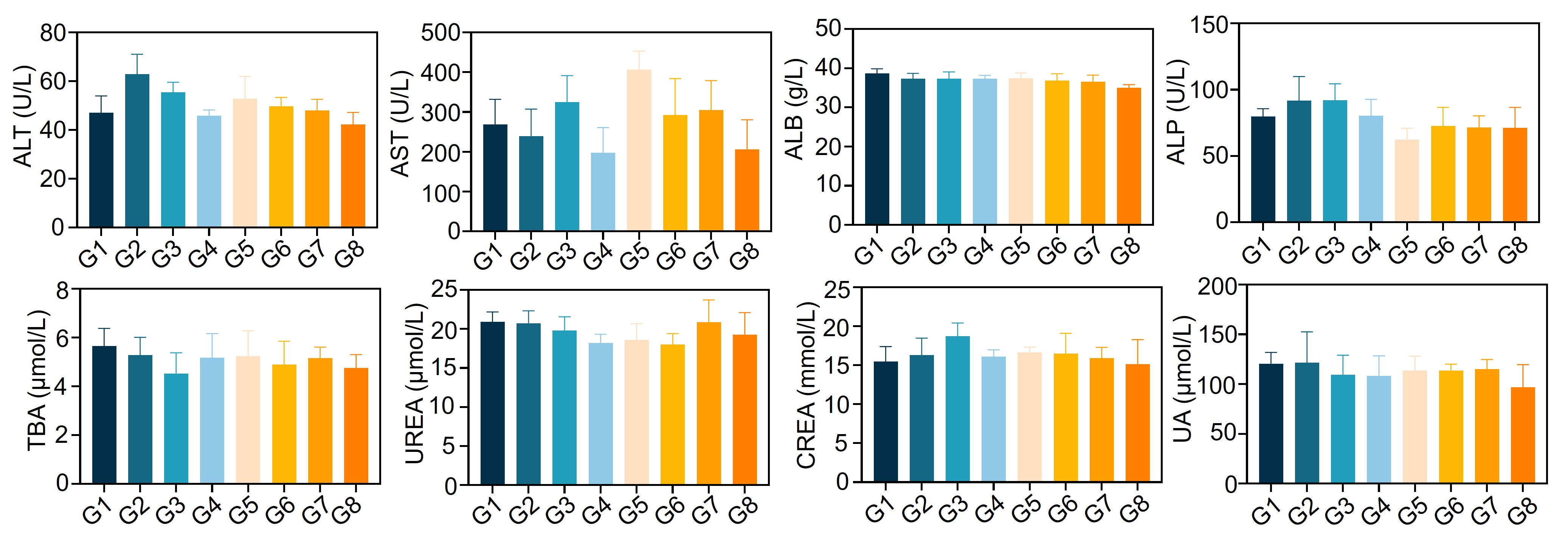
Figure S15.** Serum biochemical indexes of the mice under different treatments for 14 days. (G1. Control, G2. BP, G3. FeSe_2_, G4. BFeSe_2_, G5.US, G6. BP + US, G7. FeSe_2_ + US, G8. BFeSe_2_ + US).


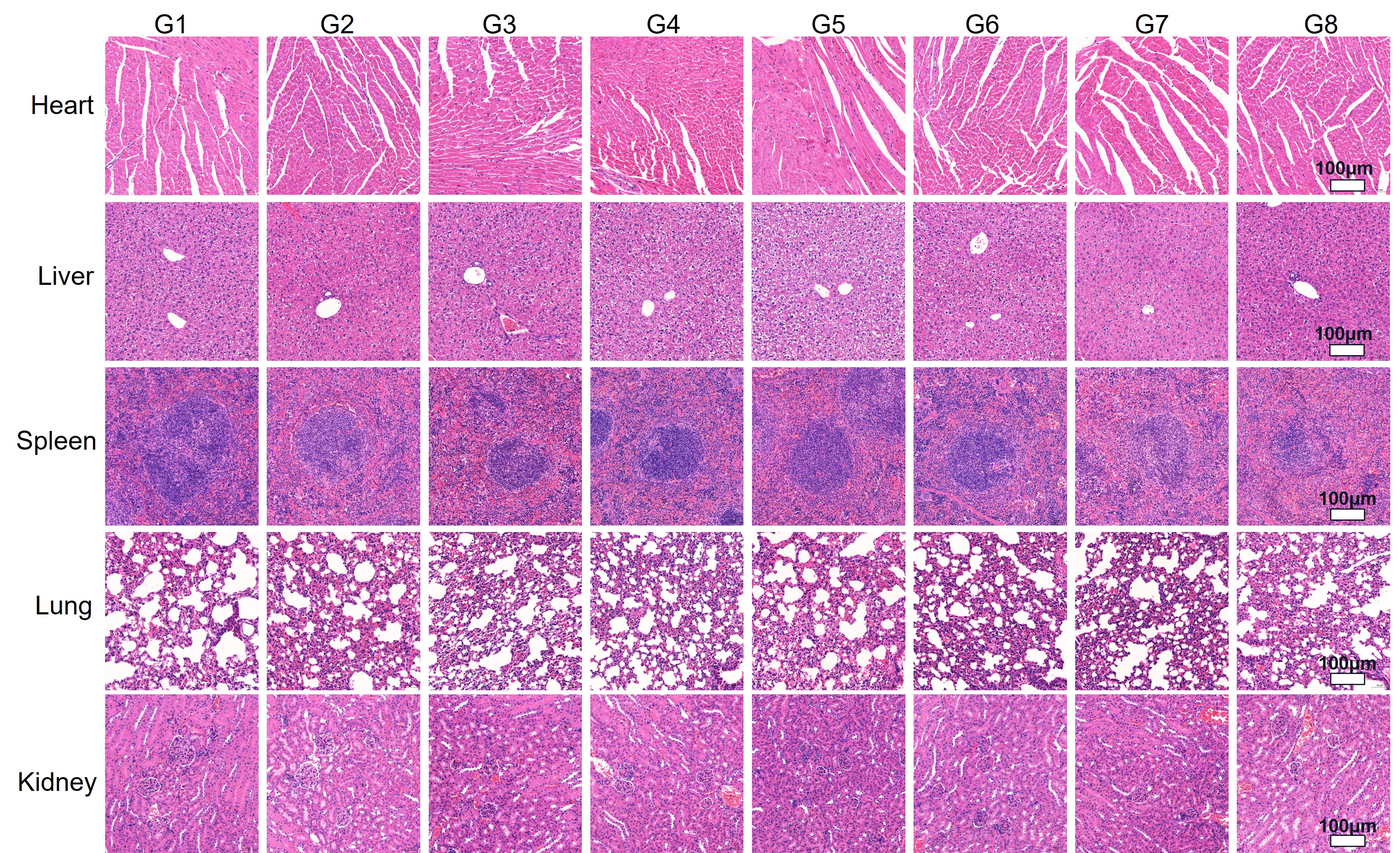


**Figure S16.** H&E staining of main organs of the mice under different treatments for 14 days. (G1. Control, G2. BP, G3. FeSe_2_, G4. BFeSe_2_, G5.US, G6. BP + US, G7. FeSe_2_ + US, G8. BFeSe_2_ + US).


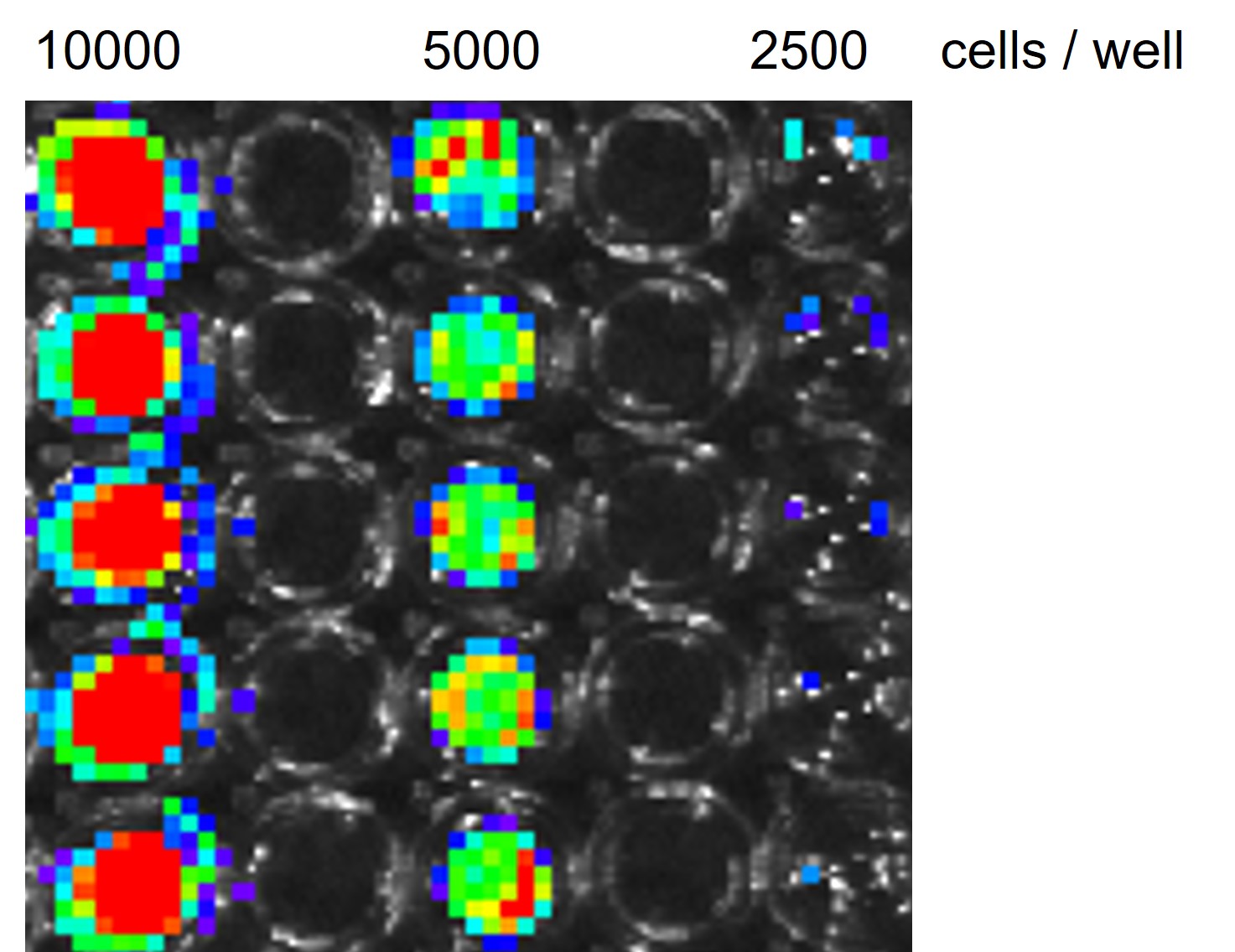


**Figure S17.** Bioluminescence images of MB49-Luc cells with different concentrations.


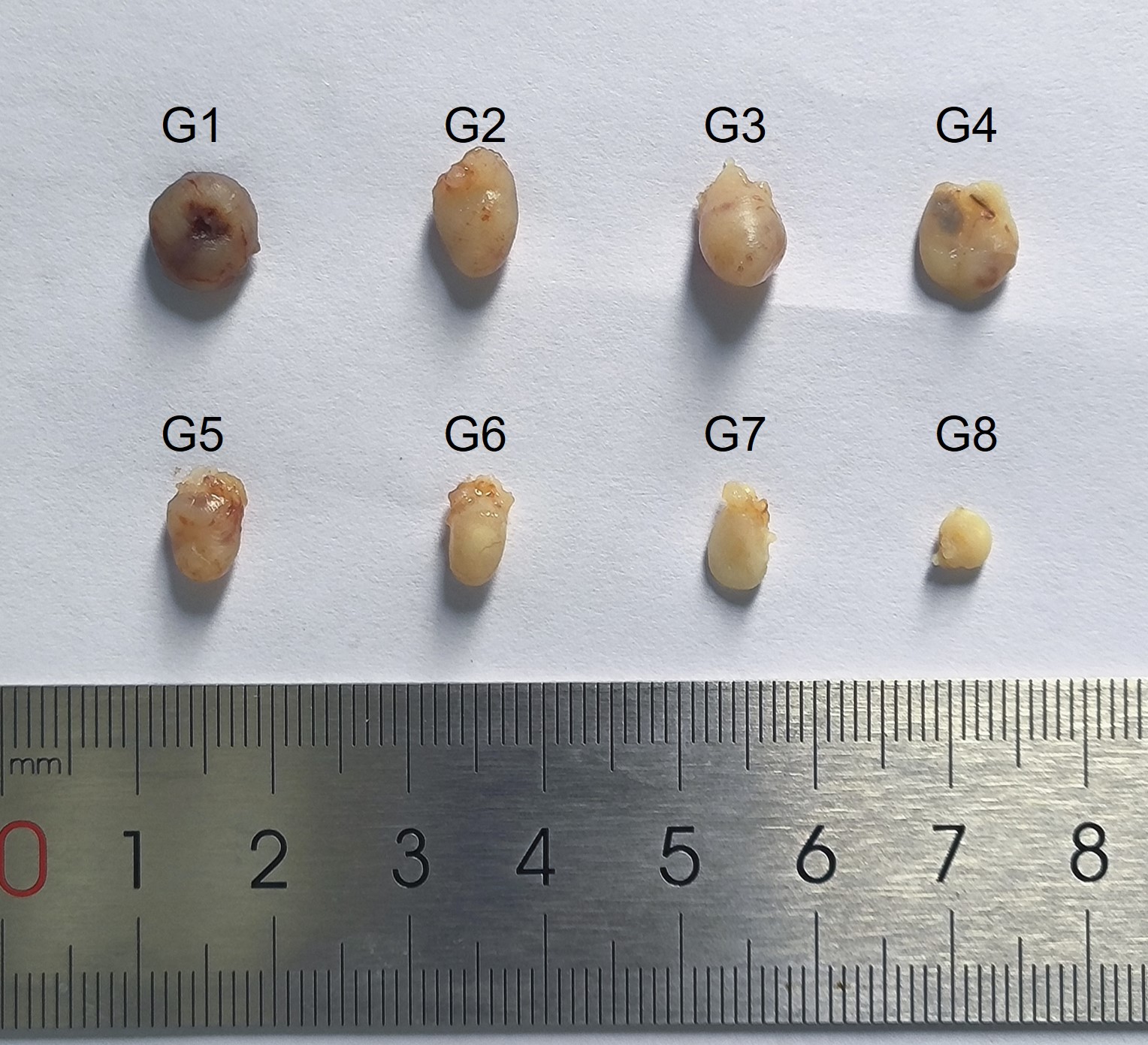


**Figure S18.** Photos of tumor in different treatments. (G1. Control, G2. BP, G3. FeSe_2_, G4. BFeSe_2_, G5.US, G6. BP + US, G7. FeSe_2_ + US, G8. BFeSe_2_ + US).
